# Comprehensive assessment of groundwater quality in the Prayagraj District, Ganga Basin

**DOI:** 10.1007/s11356-024-34030-1

**Published:** 2024-07-09

**Authors:** Bhumika Kumari, Tirumalesh Keesari, Annadasankar Roy, Hemant Mohokar, Harish Jagat Pant

**Affiliations:** 1 Isotope Hydrology Section, Isotope and Radiation Application Division, Bhabha Atomic Research Centre, Mumbai, 400 085 India; 2 Homi Bhabha National Institute, Mumbai, 400 094 India

**Keywords:** Fuzzy logic, Entropy water quality modeling, Pollution Index, Urban center, Ganga Plains, Prayagraj

## Abstract

Urbanization has severely impacted the world water resources especially the shallow groundwater systems. There is a need of a robust method for quantifying the water quality degradation, which is still a challenge for most of the urban centers across the world. In this study, a highly urbanized region of Ganga basin is selected to critically evaluate commonly used WQIs and compare with fuzzy modeling. A total of 28 water samples were collected from diverse sources (surface and groundwaters) in the vicinity of urban region covering an area of 216 km^2^ during the premonsoon period. TDS, TH, NO_3_^−^, and F^−^ values were found to be above the permissible limits in 57%, 89%, 4%, and 7% samples, respectively. The WQIs (entropy and integrated) outputs were found to be similar with 89% of the samples falling under moderate category. Fuzzy modeling was carried out allowing user-defined weighting factors for the most influential ions, and the output suggested 96% of the samples falling under moderate to excellent categories. Based on the chemical results and considering the lithology of the study area, the geochemical reactions controlling the water quality were deduced. This study outlines a systematic approach of evaluating the overall water quality of an urban region highlighting the merits and limitations of WQIs. It also justifies the immediate need to generate more robust data to achieve the sustainable development goals 6 (clean water and sanitation) and 11 (sustainability of cities and human settlement).

**Graphical Abstract:**

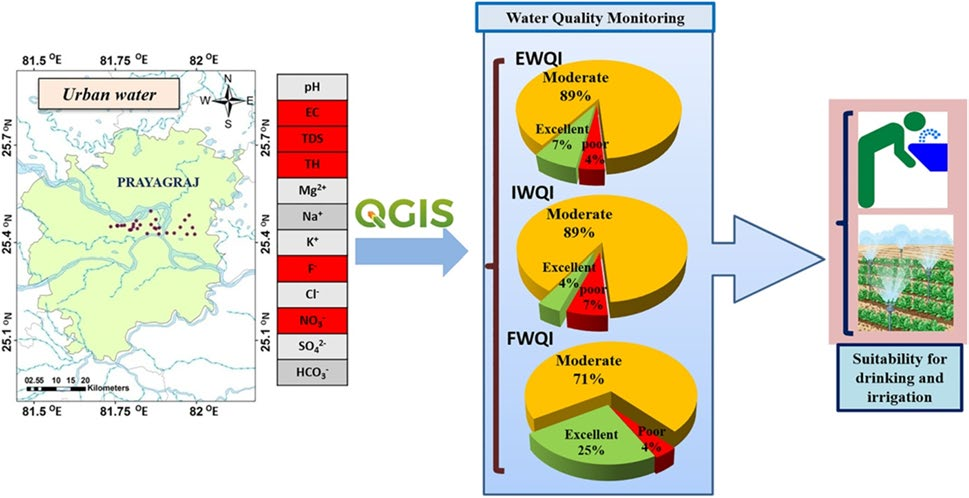

**Supplementary Information:**

The online version contains supplementary material available at 10.1007/s11356-024-34030-1.

## Introduction

Urban regions have been contributing to the rapid population growth in Ganga Plains, and the data suggest that approximately 42% of the total population in India are supported by this basin. Due to poor quality of river water and increase in population, dependence on groundwater resources for freshwater supplies has been raising continuously (Sharma et al. 2016; Mishra 2023). The groundwater resources in Ganga basin are considered essential source of good quality drinking water. However, studies reported that shallow aquifers of Middle and Deltaic Plains of Ganga basin are highly susceptible to contamination from sewage effluents; elevated levels of inorganic contaminants (F^−^, NO_3_^−^, and As), volatile organic carbons, and several kinds of pharmaceuticals pose a great risk to public health (Central pollution Control Board (CPCB) 2013; Krishan et al. 2023). Studies also indicated that prolonged intensive pumping has potential to alter natural flow regimes in Gangetic Plains and might lead to vertical migration of contaminants to greater depths > 150 m (Lapworth et al. 2018). Clearly, these factors constrain the usability of available water resources in the Gangetic Plains. Therefore, proper assessment of water quality becomes imperative for sustainable management of water resources.

An urban region belonging to Middle Ganga Plains (MGP), viz., Prayagraj district of Uttar Pradesh, India, was selected for detailed evaluation for the water quality. Previous studies have indicated that the groundwater pollution is mainly derived from anthropogenic sources like municipal wastes and industrial effluents (Central pollution Control Board (CPCB) 2013; Ghirardelli et al. 2021; Krishan et al. 2023; Richards et al. 2023). It was also observed that heavy metal residues in sediments as well as in river water are higher than the permissible limits (Pandey and Singh 2017). Microbial contamination and presence of inorganic carcinogenic elements like Cr, Cd, As, and Pb were also reported in the Ganga Plains (Maurya et al. 2019). Studies have revealed that synthetic fertilizer and industrial effluents are the main sources of nitrate in the Ganga River (Sharma et al. 2023; Balkrishna et al. 2024) and also groundwater (Madhav et al. 2018). The key indicators of water quality degradation are typically nitrate, fluoride, chloride, sodium, potassium, and dissolved oxygen (Al-Ani et al. 2019). Despite amplified anthropogenic activities and rapid industrialization, a comprehensive understanding on water quality and contamination levels in groundwater is still missing in the study area, which is partly due to compounded complexity through urbanization and continuous changes in land use and land cover.

Several Water Quality Indices (WQIs) are available in the literature with their own merits and limitations (Amiri et al. 2014; Mukate et al. 2019; Agbasi et al. 2023; Kumari et al. 2023). In this research, we have specifically used Entropy Water Quality Index (EWQI), Integrated Water Quality Index (IWQI), and Integrated Water Pollution Index (IWPI), which are well-established for obtaining overall water quality of a given area. In addition, Fuzzy Water Quality Index (FWQI) was attempted in this article, and the output is compared with the other water quality indices (WQIs). EWQI is one of the water quality probing indices, in which all the measured parameters playing a pivotal role in water are aggregated into single index (Amiri et al 2014). IWQI follows the same principle of aggregation for all the parametric concentrations into single one, but steps applied for calculation are different from other indices (Agbasi et al. 2023). Thus, in this method, new limit value is generated within the minimum and maximum acceptable guideline values. IWPI provides the clear indication of pollution loaded water and also responsible pollutant in that water (Mukate et al. 2019; Agbasi et al. 2023). NPI is a numerical index that is used to assess the risk of nitrate pollution in a given area based on factors such as soil type, climate, land use, and agricultural practices (Panneerselvam et al. 2020). The above indices are not user-defined and therefore do not allow prioritization of the contaminants based on the area of interest and user requirement. This limitation can be addressed using Fuzzy logic Water Quality Index (FWQI), which can simplify the evaluation of water quality by combining different water quality factors into a single index value considering the ambiguity and vagueness in data as well as the user defined parameters/weighting factors (Semiromi et al. 2011; Chaudhary 2020; Oladipo et al. 2021; Sajan and Christopher 2023; and Abidi et al. 2024). The use of fuzzy logic allows a more comprehensive understanding of the numerous interrelationships among water quality measurements and thus aids in the identification of pollution sources. This study is aimed at (i) appraising the overall water quality through multiple indices and (ii) identifying the most influential parameters controlling WQIs through sensitivity analysis and (iii) deducing geochemical reactions impacting water quality.

To address these objectives, we have tested the suitability of the water quality using guidelines prescribed by the BIS and WHO, followed by the estimating various indices (EWQI, IWQI, IWPI, and NPI). Finally, the water quality is evaluated using FWQI with defined steps for weight assignment and calculation. The outcome of the water quality and water pollution indices is compared, and the influencing water quality parameters are identified using sensitivity analysis. This study presents a progressive assessment of water quality from simple comparison with guideline values to estimation of quality indices to fuzzy modeling. The results from this study provide a deeper insight into the complex nature of urban hydrology as well as contribute to the development of a robust approach for monitoring the water quality in space and time.

## Study area description

**Fig. 1:**
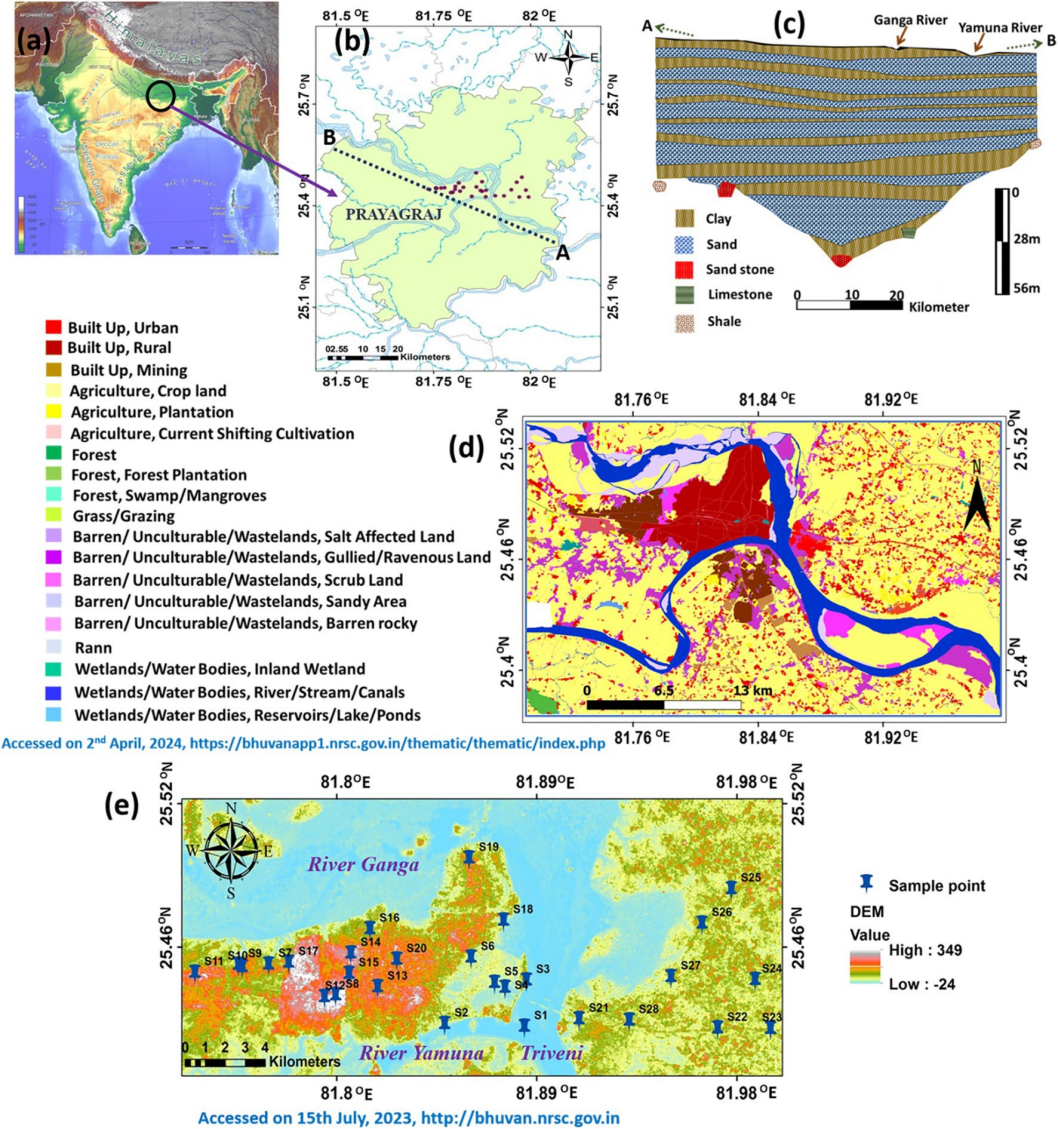
**a** Indian subcontinent, **b** Prayagraj district of Uttar Pradesh, **c** subsurface cross section along A-B transect, **d** land use and land change (LULC) map, and **e** sample locations overlaid on the digital elevation map of the study area

The study area (Prayagraj district) falls in the inter-fluvial zone of Rivers Ganga and Yamuna, located in the MGP of Uttar Pradesh (Fig. 1). This area extends between latitudes 25.42497 and 25.49625°N and longitudes 81.73613 and 81.99519°E covering an area of about 216 km^2^. The population density is about 1086/km^2^ and 25% of the population reside in urban areas. The population growth rate over the decade 2001–2011 is 20.6% (https://prayagraj.nic.in/demography). Rivers Ganga and Yamuna are the major east flowing perennial rivers in the study area that form the drainage system. Climatic condition is humid subtropical with an average rainfall of 1207 mm/year. January (9.1–23.7 °C) is the coldest month while May (27.4–42.1 °C) is the hottest month. This district is also highly irrigated due to fertile nature of the soil (Central Ground Water Board (CGWB) 2018). Depositional land formations form the most fertile lands that support rice, wheat, and sugarcane cultivation (Central Ground Water Board (CGWB) 2018). Quaternary alluvium and Vindhyan formations are the major geological units in this area. Alluvial plains sediments of Quaternary period are the most potential groundwater repositories. Alluvial deposits owe their origin to riverine activity and further subdivided into newer alluvial plains and older alluvial plains. Newer alluvial plains are confined to present day flood plain region all along the rivers while the older alluvial occupy the higher parts within the northern portions of the study area. The important land forms observed in the alluvial plain are the meanders, scrolls, point bars, back swamps, etc. The alluvial sediments are composed of sands of various grades, clay, silts, and nodular concretions of calcium carbonate at different depths. Quartz, feldspars, and ferromagnesian are the major minerals. A subsurface 2D cross section along NW–SE direction of the study area is shown in Fig. 1b. The top litho-units are composed of clay of 2 m to 20 m with minor sand layers. This clay zone is underlaid by shallow aquifer, which is continuous and extends up to 120 m. This aquifer is interspaced with minor clay beds of 2–10-m thickness. These clay and sand beds occur as alternate layers and thickness of the aquifer progressively increases towards the Rivers Ganga and Yamuna. The shallow aquifer is underlaid by a 10–15-m clay layer below which a second aquifer of very high potentiality commences and continues up to the basement (Fig. 1c). The shallow aquifer occurs under unconfined condition while deeper aquifer is under semi confined to confined conditions. The groundwater flow is towards the river Ganga in the north and river Yamuna in the south; i.e., both the rivers are effluent in nature. Both surface and groundwater resources are exploited in the study area for irrigation and domestic needs (Zakwan and Ahmad 2021). However, the gross groundwater draft for irrigation is in excess of net annual groundwater availability due to cropping pattern. The groundwater depletion is further impacted due to limited availability of surface water for irrigation. The annual fluctuation of water level rise was 0.23 to 0.74 m, and water level fall was 0.24 to 10.77 m (CGWB 2022). A land use and land cover map was prepared from the SIS-DP data downloaded from the from BHUVAN sites (https://bhuvanapp1.nrsc.gov.in/thematic/thematic/index.php) for the latest year on 2nd April 2024. The data was processed to obtain the LULC map for the study area using QGIS version 3.7 (Fig. 1d). Most of the area is covered under the agricultural land followed by urban settlements (Fig. 1d). The cartosat-1 digital elevation model was downloaded from BHUVAN (http://bhuvan.nrsc.gov.in) with tiles cdng44p and cdng44q of resolution 2.5 m accessed on 15th July 2023. The elevation data in raster format was processed and reproduced as DEM map using QGIS version 3.7 (Fig. 1e).

## Methodology

### Water sample collection

A total of 28 water samples were collected from different sources (surface water (SW)—3, hand pump (HP)—13, and tube wells (TW)—12) present in study area (Fig. 1e). Water samples were collected from the different depths for better presentation of the depth-wise variation in the hydrochemistry of study area. Water samples collected by hand pumps represent shallow aquifer (< 120 m below ground level, bgl) while tube wells represent deep aquifer. During premonsoon season (March to May), the river flows are less and hence the dependency on groundwater is high as well as its vulnerability to urban contamination. Hence, water samples were strategically collected during premonsoon period of 2015 and from sites close to human settlements, river banks, shallow, and deep groundwater and used for the analysis of WQIs. Multiparameter kit (HANNA) was used for in situ physicochemical parameters (pH, temperature, EC, and TDS). The pH probe was calibrated with standard buffer solutions (4.0, 7.0, and 10.0), and EC probe is calibrated against 0.01N KCl standard (1413 µS/cm) before field measurements. Alkalinity was measured in field by titration of 10 mL of water sample with 0.02 N H_2_SO_4_; the mixed indicator (bromocresol green–methyl red) was used for indicating the end point of the titration. About 60 mL of water sample was filtered through a cellulose filter (pore size of 0.45 µm) and transferred into a high-density polyethene (HDPE) Tarsons® bottle (APHA 1998). To avoid precipitation and adsorption of ions, C. HNO_3_ (ultra-pure) was added to the filtered samples for cation measurements. A duplicate set was collected for anion measurements and preserved without any acidification. Ion chromatography system (DX-500, Dionex Corporation) was used to measure cations (Na^+^, K^+^, Mg^2+^, and Ca^2+^) and anions (F^−^, Cl^−^, NO_3_^−^, and SO_4_^2−^) in conductivity mode with electrochemical detector (ED 40). The precision of the chemical data was verified using MERC standard solutions diluted to working range and it is found that instrumental precision is 5% RSD. Further details on chemical measurements can be found in Keesari et al. (2021). Accuracy of the chemical measurements was verified using charge balance error (CBE) shown in Eq. 1. The CBE was found to be within the allowed limits (± 5%) (Hounslow 1995).

1 $$\mathrm{CBE }\left(\mathrm{\%}\right)= \frac{\mathrm{meq}\left(\mathrm{cations}\right)-\mathrm{meq }\left(\mathrm{anions}\right)}{\mathrm{meq}\left(\mathrm{cations}\right) +\mathrm{ meq }\left(\mathrm{anions}\right)}\times 100$$

### Drinking and irrigation suitability

Water suitability for drinking is mainly evaluated by total dissolved solids (TDS) and total hardness (TH). TDS is estimated from electrical conductivity obtained in the field, and TH is estimated by adding the equivalent weights of Ca^2+^and Mg^2+^ ions, which are primarily responsible for the hardness of water (Table S1). Irrigation suitability of water is examined using different indicators such as electrical conductivity (EC), sodium adsorption ratio (SAR), residual sodium carbonate (RSC), sodium percentage (Na%), magnesium hazard (MH), corrosivity ratio (CR), and permeability index (PI). All the parameters are related to soluble salts and indicate the influence of the quantitative and compositional effects of different salts on water suitability for irrigation. The formulae for estimating these indicators are given in supplementary table (Table S1).

### Water quality indices (WQIs)

The Entropy Water Quality Index (EWQI) is an extensively used and reliable rating method for water quality estimation of any area. Biasness and uncertainty due to parametric concentration and collection of water samples from different locations can be reduced with this entropy weighted method. In the initial step, all the measured parameters for each sample is normalized using Eq. 2. Here, *Y*_*ji*_ is normalizing factor, *C*_*ji*_ is measured value of different *i*^th^ water quality parameter (WQP) of *j*^th^ groundwater samples, (*C*_*ji*_)_min_ is minimum measured value, and (*C*_*ji*_)_max_ is maximum measured value.

2 $${Y}_{ji} =\left|\frac{{C}_{ji}-{({C}_{ji})}_{\mathrm{min}}}{{\left({C}_{ji}\right)}_{\mathrm{max}}-{({C}_{ji})}_{\mathrm{min}}}\right|$$

Probability for all the *i*^th^ WQP is calculated using Eq. 3, after normalization using normalizing factors of each WQPs. *P*_*ji*_ is representing probability factor.

3 $${P}_{ji}=(1+{Y}_{ji})/{\sum }_{j=1}^{N}(1+{Y}_{ji})$$

In the next step, probability factor (*P*_*ji*_) of WQP is used for the calculation of information entropy (*e*_*i*_) of WQP (Eq. 4), and *N* is number of samples collected.

4 $$e_i=-\frac1{ln(N)}\sum\nolimits_{j=1}^NP_{ji}\;\mathit{ln}(P_{ji})$$

Relative weight (*W*_*i*_) is calculated and assigned for each parameter. On the basis of values of *W*_*i*_, each parameter is divided into two categories; semi-critical (0.05–0.1) and critical (> 0.1). Weight (*w*_*i*_) is calculated for each *i*^th^ WQPs using Eq. 5. Then, relative weight is calculated using *w*_*i*_ and expressed by *W*_*i*_ (Eq. 6).

5 $${w}_{i} = (1-{ e}_{i})$$

6 $${W}_{i}={w}_{i}/{\sum }_{j=1}^{N}{w}_{i}$$

Quality rating (*q*_*i*_) of *i*^th^ WQP is calculated using Eq. 7, and for pH, Eq. 8 is used. In Eq. 7, *C*_*i*_ is measured value of *i*^th^ WQPs and *SD*_*i*_ is for standard of WQPs. In Eq. 8, *C*_*pH*_ is measured value of pH for water samples while *S*_*pH*_ is standard pH. In this study, the standard is considered as per WHO (2011) and BIS (Bureau of Indian Standards) (2012).

7 $$qi= \left(Ci/SDi\right)\times 100$$

8 $$qi= ({C}_{pH}-7)/({S}_{pH}-7)$$

EWQI is calculated using Eq. 9, inputting the values of quality rating (*q*_*i*_) and relative weight (*W*_*i*_).

9 $$\mathrm{EWQI}=\sum\nolimits_{i=1}^{n}{W}_{i }. {q}_{i}$$

Based on calculated EWQI, water quality can be classified into excellent (EWQI < 25), good (26–50), moderate (51–75), poor (76–100), and extremely poor (> 100) categories.

In the case of Integrated Water Quality Index (IWQI), the subindex (SI) for each WQPs is calculated and then all the computed SIs are added to obtain a single IWQI score (Mukate et al. 2019). The modified permissible limits (MoPL) for each individual WQPs are calculated based on the desirable limit (DL) and maximum permissible limit (MPL) set by BIS (Bureau of Indian Standards) (2012). Parameter with both DL and maximum permissible limit MPL is called relaxable and with only one limit value is called non-relaxable. MoPL for the relaxable parameter is calculated using Eq. 10. The 20% of range is calculated using Eq. 11.

10 $$\text{MoPL}=\text{maximum permissible limit }\left(\text{MPL}\right)- (20\;\mathrm{\%}\;\text{of range})$$

11 $$\mathrm{range}=\text{maximum permissible limit }\left(\mathrm{MPL}\right)-\text{desirable limit }(\mathrm{DL})$$

In the absence of DL, MoPL for non-relaxable parameters is calculated using Eq. 12.

12 $$\text{MoPL}=\text{PL}-(20\;\mathrm{\%}\;\text{of the PL})$$

In Eqs. 10 and 12, 20% of the range of the parameter is selected as an alert or threshold level of the pollution under which aquifer can be restored or revived. This limit, 20% in this case, is flexible and can be modified based on the contaminant (Mukate et al. 2019). All the calculated DL, MPL, and MoPL are categorized into pre-defined ranges, and by comparing these calculated values with respect to *P*_*i*_ value, i.e., measured parametric value of sample, SI values are assigned.

Case 1: DL ≤ *P*_*i*_ ≥ MoPL. When *P*_*i*_ lies between DL and MoPL, the SI_1_ is zero (${\mathrm{SI}}_{1}=0$).

Case 2: *P*_*i*_ ≤ DL. When *P*_*i*_ is less than or equal to desirable limit, SI_2_ is calculated using Eq. 13.

13 $${\mathrm{SI}}_{2}= \frac{\mathrm{DL}-{P}_{i}}{\mathrm{DL}}$$

Case 3: *P*_*i*_ ≥ MoPL. When the MoPL is equal and less than *P*_*i*_, SI_3_ is calculated using Eq. 14.

14 $${\mathrm{SI}}_{3}= \frac{{P}_{i}-\mathrm{MoPL}}{\mathrm{MoPL}}$$

After categorization and calculation of each SI, IWQI score is computed by summing all SIs (Eq. 15).

15 $${\mathrm{IWQI}}_{i}= \sum\nolimits_{i=1}^{n}{\mathrm{SI}}_{ij}$$

According to different IWQI values, water quality is divided into different classes, viz., Excellent (< 1), Good (1–2), Marginal (2–3), Poor (3–5), and Unsuitable (> 5).

### Water pollution indices

The Water Pollution Index (WPI) is used to appraise the degree of pollution in water bodies. WPI can be used for large number of samples for both relaxable and non-relaxable parameters. Traditionally used water quality indicators can be utilized for estimation of pollution (Amuah et al. 2022). In this study, measured hydro-chemical parameters of water samples are examined with their BIS limits using Integrated Water Pollution Index (IWPI), which is developed by Hossain and Patra (2020). The degree of pollution by a specific pollutant impacting the water quality is determined using pollution load (PL). PL is measurement of stress placed upon an aquatic ecosystem due to pollutants. PL is calculated using measured values (*I*_*c*_) of each parameter and standard limits (*S*_*d*_) set by BIS (Eq. 16).

16 $$\mathrm{PL}=1+\left[\frac{{{I}_{c}-S}_{d}}{{S}_{d}}\right]$$

All the calculated PL values of parameters are integrated and transformed into a single value i.e., IWPI (Eq. 17).

17 $$\mathrm{IWPI}= \frac{1}{n}\sum\nolimits_{i=1}^{n}\mathrm{PL}$$

Based on calculated IWPI values, water quality is divided into four categories viz., excellent (< 0.5), good (0.5–0.75), moderately polluted (0.75–1), and highly polluted water (> 1).

Considering the widespread groundwater nitrate pollution and its impact on human health, Nitrate Pollution Index (NPI) is calculated and implemented in this study. The parameter *C*_*m*_ is defined as the measured concentration of nitrate in collected water samples and *C*_*s*_ is the threshold value of nitrate contamination resulting from anthropogenic pollution. Both *C*_*m*_ and *C*_*s*_ are used for the calculation of NPI (Panneerselvam et al. 2020) (Eq. 18).

18 $$\mathrm{NPI}= \frac{{C}_{m}- {C}_{s}}{{C}_{s}}$$

Based on NPI values, water quality can be divided into five categories: no pollution (< 0), light pollution (0–1), moderate pollution (1–2), significant pollution (2–3), and very significant pollution (> 3).

### Fuzzy water quality index

Fuzzy Water Quality Index (FWQI) is a versatile methodology utilized in the field of environmental research and water quality monitoring. FWQI provides better flexibility to the evaluation process by accounting for the ambiguous or imprecise water quality data, which is not offered by conventional WQIs. Fuzzy logic techniques are employed in FWQI to describe water quality scenarios more accurately, and it also considers variables that could be challenging to quantify using traditional techniques. A brief overview of FWQI estimation is provided below.

#### Fuzzy inference rules

In case of fuzzy inference system, IF–THEN rules along with fuzzy set operations are employed to use the relationship between input and output parameters of a certain system. The detailed rules used in the current study are presented in Table 1. Basic construction of a fuzzy inference system (FIS) is given below.

**Table 1:** IF–THEN rule for assignment of weight for each water samples (Sajan and Christopher 2023; Oladipo et al. 2021)

Rule No	Fuzzy rules	Weight
1	IF (pH is medium) AND (TDS is low) AND (F^−^ is low) AND (Cl^−^ is low) AND (NO_3_- is low) AND (SO_4_^2−^ is low) AND (Mg^2+^ is low) AND (Ca^2+^ is low) AND (TH is low) THEN (WQ is Excellent)	1
2	IF (pH is medium) AND (TDS is medium) AND (F^−^ is medium) AND (Cl^−^ is medium) AND (NO_3_^−^ is medium) AND (SO_4_^2−^ is medium) AND (Mg^2+^ is medium) AND (Ca^2+^ is medium) AND (TH is medium) THEN (WQ is Moderate)	0.5
3	IF (TDS is medium) OR (F^−^ is medium) OR (NO_3_^−^ is medium) OR (TH is medium) THEN (WQ is Moderate)	0.5
4	IF (pH is medium) AND (TDS is high) AND (F^−^ is high) AND (Cl^−^ is high) AND (NO_3_^−^ is high) AND (SO_4_^2−^ is high) AND (Mg^2+^ is high) AND (Ca^2+^ is high) AND (TH is high) THEN (WQ is Poor)	0.25
5	IF (F^−^ is high) OR (NO_3_^−^ is high) OR (TH is high) THEN (WQ is Poor)	0.25
6	IF (Mg^2+^ is high) AND (Ca^2+^ is high) AND (TH is high) THEN (WQ is Poor)	0.25
7	IF (TDS is high) THEN (WQ is Poor)	0.25

*AND = min approach; OR = max approach; Implication = min; Aggregation = max; Defuzzification = centroid; Mamdani approach

The FIS involves main four steps and is mainly based on fuzzy set theory (Ross 1977). The steps are as follows:

- Fuzzification: This step involves formulation of membership functions to be used for the conversion of crisp values of input parameters into respective linguistic terminologies. The assumed ranges of the different subclasses of the concerned parameters are further used to identify the membership function value for different parameters. In the current study triangular membership functions were used and the description of the functions are provided in Table 2.
- Evaluation of the fuzzy rules: In this step compounding of the outcome from fuzzy IF–THEN rule is performed. During this process, the class of fuzzy index for a specific rule is determined by selecting the lowest class among all the chosen parameters. Moreover, in this step, the class and corresponding value of membership function with the lowest position among the selected parameters become the fuzzy index class for the rule. It involves identification of the applicable fuzzy rules for the given data.
- Aggregating rule outputs: In this step, the algorithm considers the applicable fuzzy IF–THEN rules. It identifies the maximum value of the fuzzy index for the same class among all the available applicable rules. The outputs of all these relevant rules are then combined into a single fuzzy distribution by carrying out the fuzzy union operation. This approach enables the algorithm to efficiently aggregate the outcome of the applicable rules and create a comprehensive representation of the fuzzy distribution.
- Defuzzification: Defuzzification involves transforming the fuzzified output into a value using various defuzzification methods.

**Table 2:** Triangular membership functions used for fuzzification

Triangular membership functions				
Parameter	Low	Medium	High	Range
	[*a b c*]	[*a b c*]	[*a b c*]	
pH	[0 0 6.5]	[6.5 7.5 8.5]	[8.5 14 14]	[0 14]
TDS	[0 0 500]	[500 750 1000]	[1000 1500 1500]	[0 1500]
F^−^	[0 0 0.5]	[0.5 1 1.5]	[1.5 2 2]	[0 2]
Cl^−^	[0 0 125]	[125 187.5 250]	[250 500 500]	[0 500]
NO_3_^−^	[0 0 25]	[25 37.5 50]	[50 100 100]	[0 100]
SO_4_^2−^	[0 0 125]	[125 187.5 250]	[250 500 500]	[0 500]
Mg^2+^	[0 0 50]	[50 75 100]	[100 200 200]	[0 200]
Ca^2+^	[0 0 100]	[100 200 300]	[300 400 400]	[0 400]
TH	[0 0 250]	[240 370 500]	[490 1000 1000]	[0 1000]
FWQI	[0 0 45]	[35 55 75]	[65 100 100]	[0 100]

For this current study, Mamdani FIS algorithm (Mamdani and Assilian 1975) was used in MATLAB v.2016a software (https://www.mathworks.com/help/fuzzy/types-of-fuzzy-inference-systems.html). The “min” implication along with “max” aggregation and “centroid” defuzzification approaches were employed to estimate the final FWQI.

### Sensitivity analysis

Sensitivity analysis is used to understand the rational importance and the role of parameters in calculation of water quality indices (Lodwick et al. 1990). In this study, the sensitivity analysis was carried out for EWQI, IWQI, and IWPI. To calculate the sensitivity values, a revised water quality index was estimated by removing one input parameter each time (Eq. 19). The average sensitivity values of each input water quality parameter are estimated using Eq. 20.

19 $${S}_{i}= \frac{\left|\frac{{V}_{i}}{N}-\left.\frac{{v}_{i}}{n}\right|\right.}{{N}_{i}}$$

20 $${S}_{avg}=\frac{\sum_{i=1}^{m}{S}_{i}}{m}$$

In the above equations, *S*_*i*_ is the sensitivity value of indices after removing *i*^th^ parameter, *V*_*i*_ is the index score of *i*^th^ groundwater sample, and *v*_*i*_ is the index score after *i*^th^ parameter removed. *N* is total number of parameters, and *n* is number of parameters after removal, with both *N* and *n* used for the calculation of *V*_*i*_ and *v*_*i*_, respectively. *S*_*avg*_ is average sensitivity values of *i*^th^ parameter, and *m* is total number of water samples.

## Results

### Physicochemical parameters

**Fig. 2:**
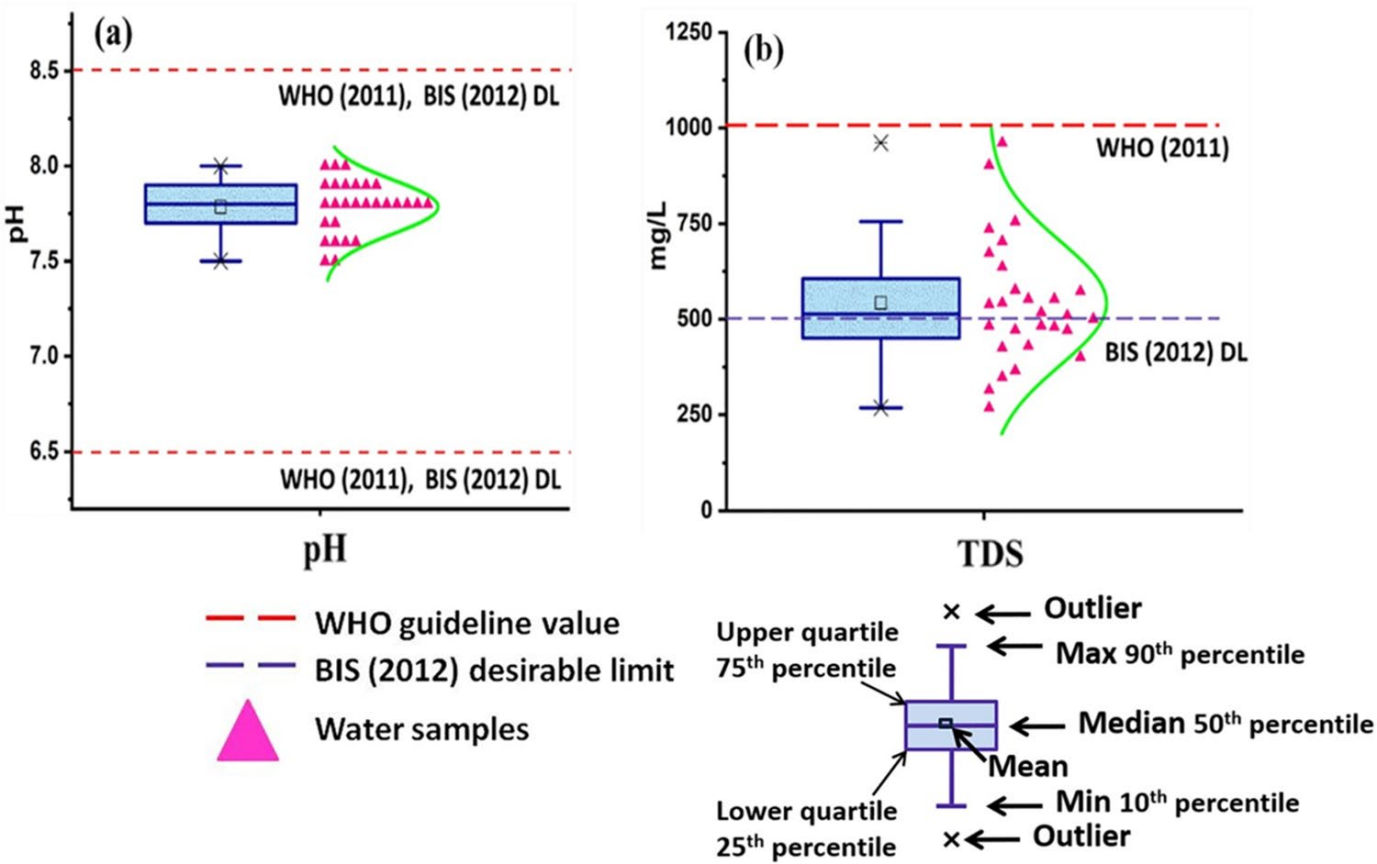
Box-whisker plots of **a** pH and **b** TDS along with distribution curves. Guideline values are indicated by dash lines where applicable

pH is the most important indicator of the water health. pH value < 7 represents acidic nature of water while > 7, basic nature of water. In the study area, pH is in the range of 7.5 to 8 (mean: 7.79), and all the water samples fall under acceptable range of 6.5–8.5 set by both WHO (2011) and BIS (Bureau of Indian Standards) (2012) during premonsoon (Fig. 2a, Table 3, Table S2). Temperature of the water samples ranged from 27.8 to 34.1 °C with an average value of 29.8 °C. EC is another important parameter used for evaluating the potability of water samples. EC measures the concentration of dissolved ions and salts based upon conductivity. EC values of the water samples range from 400 to 1435 µS/cm with an average value of 809 µS/cm. TDS represents inorganic salts of calcium, magnesium, potassium, sodium, bicarbonates, chlorides, and sulfates and some minor amounts of dissolved organic matter (WHO 2011). TDS in the water samples ranges from 268 to 961 mg/L with an average value of 542 mg/L (Fig. 2b, Table S2). High TDS alters the taste and hardness of water and renders water unsuitable for drinking (WHO 2011). TDS values of the collected water samples are within the permissible limit of 1000 mg/L (WHO 2011) suggesting that waters are suitable for potable purpose but about 57% of water samples fall between desirable and permissible limits as per BIS (Bureau of Indian Standards) (2012) (Table 3). Nine HP and seven TW samples showed TDS values above DL of BIS (Bureau of Indian Standards) (2012), i.e., 500 mg/L.

**Table 3:** Percentage of samples exceeding the BIS (Bureau of Indian Standards) (2012) and WHO (2011) levels and related health hazards

Parameters	WHO (2011)	BIS (Bureau of Indian Standards) (2012)		% of samples exceeding WHO (2011)	% of samples exceeding BIS (Bureau of Indian Standards) (2012) DL	% of samples exceeding BIS (Bureau of Indian Standards) (2012) MPL	Human health related concerns based on parameters
	Guideline value	DL	MPL				
pH	6.5–8.5	6.5–8.5	No relaxation	0.0	0.0	0	Ingestion—stomach upset, taste change Dermatological—skin dryness and itchiness
TH (as CaCO_3_)	500	200	600	0.0	89.0	0	Ingestion—kidney stone, heart related disease, calcification at arteries
TDS	1000	500	2000	0.0	57.0	0	Ingestion—constipation, laxative effect on body
Ca^2+^	300	75	200	0.0	18.0	0	Ingestion—rickets (low concentration), kidney disease and stone in bladders (high concentration)
Mg^2+^	100	30	100	0.0	64.0	0	Ingestion—cathartic and diuretic
F^−^	1.5	1	1.5	0.0	7.0	0	Ingestion—dental fluorosis and skeletal fluorosis (excessive concentration)
NO_3_^−^	50	45	No relaxation	4.0	4.0	0	Ingestion—blue baby syndrome (lactating mother to infant)
Cl^−^	250	250	1000	0.0	0	0	Ingestion—gastrointestinal irritation, bitter and unpleasant taste of water (Exceeded concentration of 250 mg/L)
SO_4_^2−^	250	200	400	0.0	0.0	0	Ingestion—kidney disease, dehydration

*Unit of all parameters are in mg/L, except pH

### Major ion chemistry

#### Cations

**Fig. 3:**
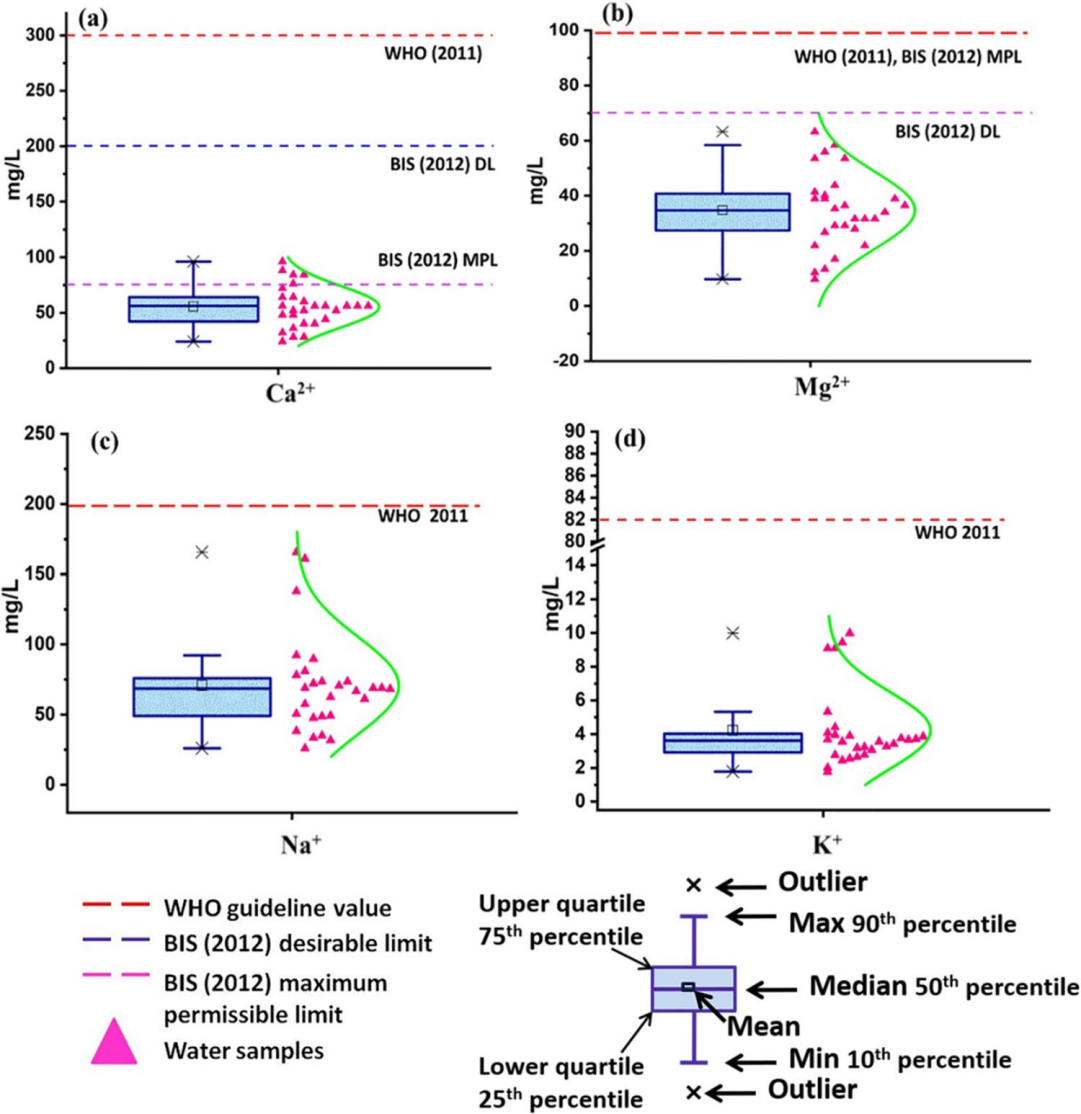
Box-whisker plots of **a** Ca^2+^, **b** Mg^2+^, **c** Na^+^, and **d** K^+^ along with distribution curves. Guideline values are indicated by dash lines where applicable

Alkali (Na^+^ and K^+^) and alkaline earth metal ions (Ca^2+^ and Mg^2+^) help in interpreting the geochemical nature of water as well as in assessing the quality of water. In study area, Ca^2+^ is found to vary from 24 to 96.2 mg/L with an average value of 55.4 mg/L (Table S2) during premonsoon. About 18% of the samples exceed the desirable limit (DL) set by BIS (Bureau of Indian Standards) (2012) i.e., 75 mg/L. But, none of the samples shows Ca^2+^concentration above WHO (2011) and BIS (Bureau of Indian Standards) (2012) maximum permissible limits, i.e., 300 mg/L and 200 mg/L, respectively (Fig. 3a, Table 3). Four shallow and one deep groundwater samples showed Ca^2+^ concentration above the desirable limit set by BIS (Bureau of Indian Standards) (2012). Ca^2+^ in groundwater is mainly contributed to weathering of silicates and carbonates. Consumption of Ca^2+^ rich water may result in problem related to urinary passage, increase the vulnerability of kidney or bladder stone. On the contrary, consumption of Ca^2+^ deficient water causes rickets (WHO 2011). The Mg^2+^ content of water samples ranges from 9.73 to 63.2 mg/L (mean value: 34.7 mg/L) and falls within the permissible limit set by WHO (2011) and BIS (Bureau of Indian Standards) (2012) (MPL) (Table S2). But, based on the desirable limit set by BIS (Bureau of Indian Standards) (2012), about 64% of the samples are not suitable for potable purposes (Fig. 3b, Table 3). Nine samples of each shallow and deep groundwater showed Mg^2+^concentration above desirable limit set by BIS (Bureau of Indian Standards) (2012). Biochemically, Mg^2+^ is very essential ion because of its presence in the core site of chlorophyll and enzymatic activity. High Mg^2+^ causes cathartic and diuretic effects in human (WHO 2011).

Na^+^ and K^+^ concentrations in the water samples range from 25.9 to 166 mg/L (mean value: 70.7 mg/L) and 1.78 to 10 mg/L (mean value: 4.22 mg/L) respectively (Fig. 3c, d, Table S2). Silicate rocks are common sources of Na^+^ and K^+^ in groundwater. No health-based limit is prescribed by WHO for Na^+^, but concentration greater than 200 mg/L may exhibit aesthetic effect, like, poor taste of drinking water (WHO 2011). Similarly, there is no health-based limit for K^+^ in water (BIS (Bureau of Indian Standards) 2012), but reports suggest that consumption of K^+^ (82 mg/L) rich water may lead to nervousness and abdominal malfunctioning (WHO 2011).

#### Anions

**Fig. 4:**
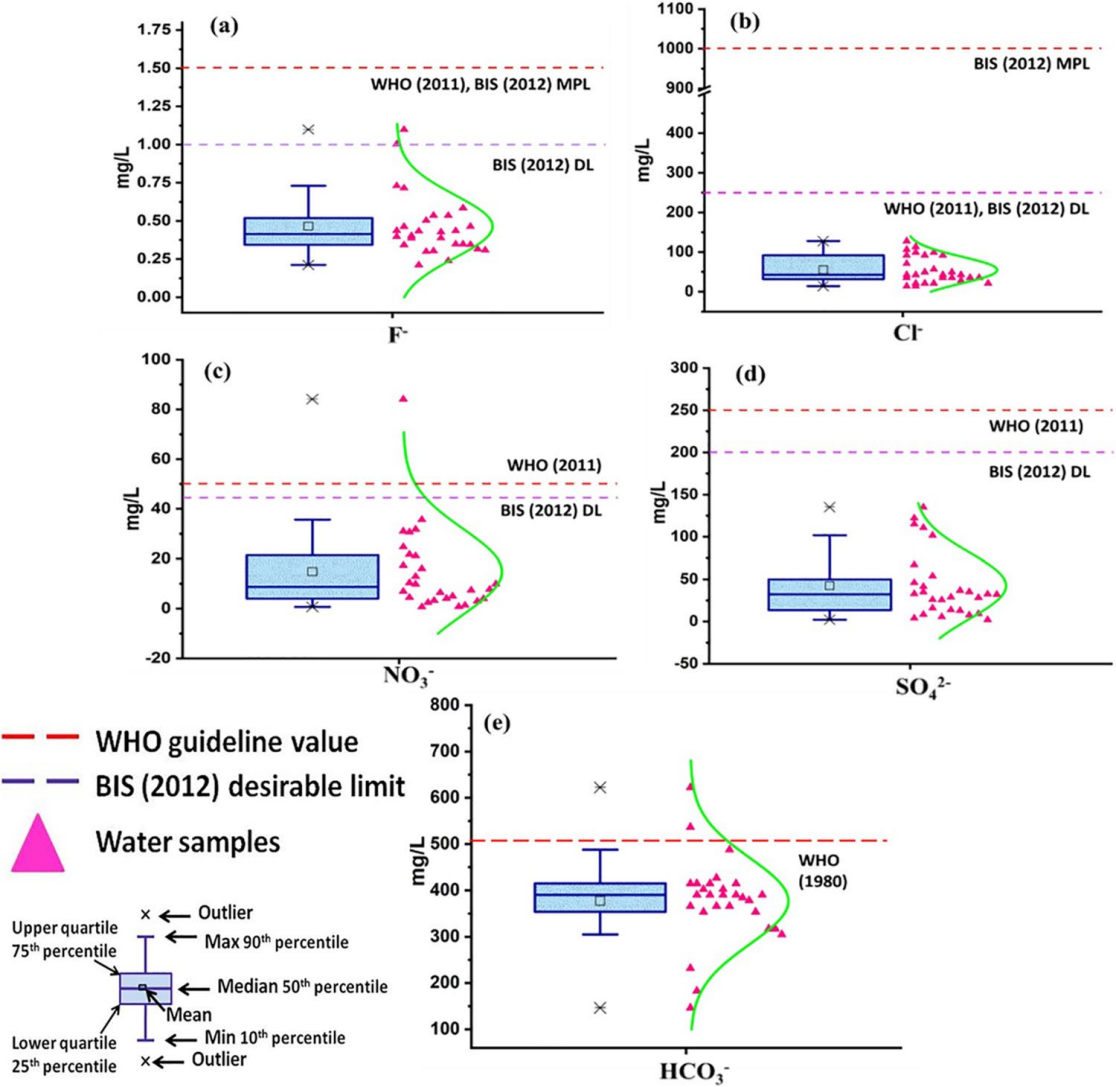
Box-whisker plots of **a** F^−^, **b** Cl^−^, **c** NO_3_^−^, **d** SO_4_^2−^, and **e** HCO_3_^−^ along with distribution curves. Guideline values are indicated by dash lines where applicable

Among anions, F^−^ and NO_3_^−^ are very common contaminants in various water bodies in India and abroad (Central pollution Control Board (CPCB) 2013; Su et al. 2021). In the study area, F^−^concentration ranges from 0.21 to 1.1 mg/L (mean value: 0.464 mg/L) and all the samples fall within the permissible limits set by WHO (2011) and BIS (Bureau of Indian Standards) (2012) (Fig. 4a, Table 3, Table S2). But, two samples (S-13 and S-24) show F^−^concentration above the desirable limit of BIS (Bureau of Indian Standards) (2012) (Table S2). Fluoride (< 0.6 mg/L) deficient water may promote tooth decay, while high concentration (> 1.5 mg/L) may lead to dental and skeletal fluorosis. There are no evidences of physiological manifestations of fluoride related diseases such as dental and skeleton fluorosis in local population of this area (Pandey et al. 2020). NO_3_^−^ concentration in water samples ranges from 0.71 to 84.1 mg/L with an average value of 14.8 mg/L (Fig. 4c, Table S2). Only one water sample (S-25) collected from shallow aquifer falls above the desirable limits of BIS (Bureau of Indian Standards) (2012) while rest of the samples show NO_3_^−^ concentration within permissible limits set by BIS (Bureau of Indian Standards) (2012) and WHO (2011) (Table 3, Table S2). Nitrate related health hazard is generally observed in infants, known as blue-baby syndrome (methemoglobinemia), which affects the oxygen carrying capacity of the blood.

Cl^−^ concentration in water samples range from 14.2 to 128 mg/L (mean value: 55 mg/L) and SO_4_^2−^concentration from 2.19 to 135 mg/L (mean value: 42.4 mg/L) (Fig. 4b, d, Table S2). Both Cl^−^ and SO_4_^2−^concentrations of water samples fall within acceptable limits (Table 3). High concentration of Cl^−^ in water inhibits the microbial activity and consumption may cause laxative effect. Consumption of SO_4_^2−^ rich water may promote dehydration, cathartic effect, and unpleasant taste of water. HCO_3_^−^ concentration in water samples of the study area ranges from 142–622 mg/L with an average value of 377 mg/L. Based on the guideline value of 500 mg/L for HCO_3_^−^ (WHO 2011), two samples (S-5 and S-18) show higher HCO_3_^−^ values while rest of the samples fall within the limits (Fig. 4e, Table S2). High alkalinity affects physical properties of water and harms aquatic life (WHO 2011).

### Assessment of water quality for irrigation

In this study, only specific and significant indicators are applied for evaluating the suitability of water for irrigation. Indicators used are classified from excellent to poor classes for better illustration of the quality level. EC is basic and mostly applied indicator as it is linked to the concentration of dissolved ions, which influences the soil health and plants growth. On the basis of EC, irrigation suitability of water is grouped into five classes, viz., excellent class (EC < 250 µS/cm), good (250–750 µS/cm), permissible (750–2000µS/cm), doubtful (2000–3000 µS/cm), and unsuitable (> 3000 µS/cm). Excellent class indicates that water is most suitable and containing optimum number of ions essential for growth of crops, permissible class indicates the slight diversion of quality of water from its irrigation suitability but can be utilized by mixing with freshwater. Doubtful class indicates treatment of water is needed before using for agriculture while unsuitable class indicates that natural quality of water is irreversible and cannot be used for agricultural purposes. About 46% and 54% of samples fall under good and permissible classes respectively (Table 4). None of the samples falls under excellent or unsuitable classes. Therefore, it can be considered that most of the water samples of study area are permissible for irrigational use. A few samples with high EC (> 750 µS/cm) may not be suitable for growth of sensitive plants (Bauder et al. 2011).

**Table 4:** Qualitative classification of irrigation water based on different parameters

Ranges	Classification of water based on parameters	% of samples
EC (µs/cm)		
< 250	Excellent	0
250–750	Good	46
750–2000	Permissible	54
2000–3000	Doubtful	0
> 3000	Unsuitable	0
Alkalinity hazard (SAR)		
< 10	Excellent	100
18–10	Good	0
18- 26	Doubtful	0
> 26	Unsuitable	0
Percent sodium (%Na)		
< 20	Excellent	7
20–40	Good	64
40–60	Permissible	29
60–80	Doubtful	0
> 80	Unsafe	0
Residual sodium carbonate (SAR)		
< 1.25	Good	71
1.25–2.5	Doubtful	29
> 2.5	Unsuitable	0
Magnesium hazard (MH)		
< 50	Suitable	43
> 50	unsuitable	57
Chloride toxicity		
< 70	Safe for all plants	68
70–140	Sensitive plants usually show slight to moderate injury	32
141–350	Moderately tolerant plants usually show slight to substantial injury	0
> 350	can cause severe problem	0
Corrosivity ratio (CR)		
< 1	Suitable for pipe	100
> 1	Unsuitable for pipe	0

SAR, RSC and Na% are directly related to the concentration of Na^+^ ion. Na^+^ ion replaces Ca^2+^ and Mg^2+^ ions and combines with HCO_3_^−^ and CO_3_^−^ and culminate into compaction and non-permeability of soil which, leads to death of plants and crops. SAR values of the water samples range from 0.75 to 3.8 (mean value: 1.86). Based on SAR values water quality is divided into four classes, viz., excellent (< 10), good (10–18), doubtful (18–26), and unsuitable (> 26). Excellent class defines most suitable water for irrigation, good class indicates suitable water but excessive use of fertilizers may deviate its natural quality. Doubtful class means that use of the water either affects the soil fertility by sodication and compaction. This water can be used for irrigation after treatment or dilution with freshwater. Unsuitability means that water is not fit for irrigation. It is observed that all the water samples fall in the excellent class (100%) (Table 4). RSC value of the water ranges from − 2.6 to 2.2 meq/L (mean value: 0.56 meq/L). Based on RSC value, 71% of samples fall under good category (RSC value < 1.25) and 29% under doubtful category (RSC 1.25–2.5) and these samples might inflict soil sodication (Table 4). Na% value ranges from 17.6 to 55.6 with an average value of 35.9. Water samples show a wide range of quality classes based on the Na%. About 64% and 29% of samples fall under good (20–40) and permissible (40–60) classes respectively while 7% samples fall under excellent (< 20) category (Table 4). Based on Na% values, majority of the samples are fit for irrigation purpose.

Balanced amount of magnesium is necessary for crop growth, but higher magnesium in irrigation water may affect the physical structure of land, infiltration, crop yield and biotic components negatively. Land degradation, draught, and low productivity are some irreversible hazards caused by excessive amount of magnesium in water. MH provides the level of hazard correlated to the magnesium containing irrigational water and their impact on the crops, soil and the topography. MH values of the water are found to be 20.8–70.2 (mean value: 50.1). Based on MH values, 43% of the samples are found to be suitable which means the amount of magnesium is balanced by other ions while 57% are unsuitable indicating magnesium is higher than the optimum levels (Table 4). Although Cl^−^ is necessary for plant growth, excess chloride in irrigation water may be toxic to crops and hamper agricultural production (Bauder et al. 2011). In this study, 68% of samples are found to be safe for plant growth (Cl^−^  < 70 mg/L), and the remaining 32% pose moderate injury to plants (Cl^−^: 70–140 mg/L) (Table 4). Presence of salts and ions in water may facilitate oxidation–reduction reactions in the metallic pipes causing deteriorations of its quality and durability. Hence, CR is used to qualify water for metal piping and other applications. CR values of the water samples range from 0.082 to 0.768 (mean value: 0.34) and all the water samples (100%) show CR < 1, hence suitable for metallic piping applications (Table 4). CR suitability indicates the absence of potential reactions among the ions present in the water and metal groups present in the metallic pipe. Durability of metallic pipe will not get affected by waters of this area.

**Fig. 5:**
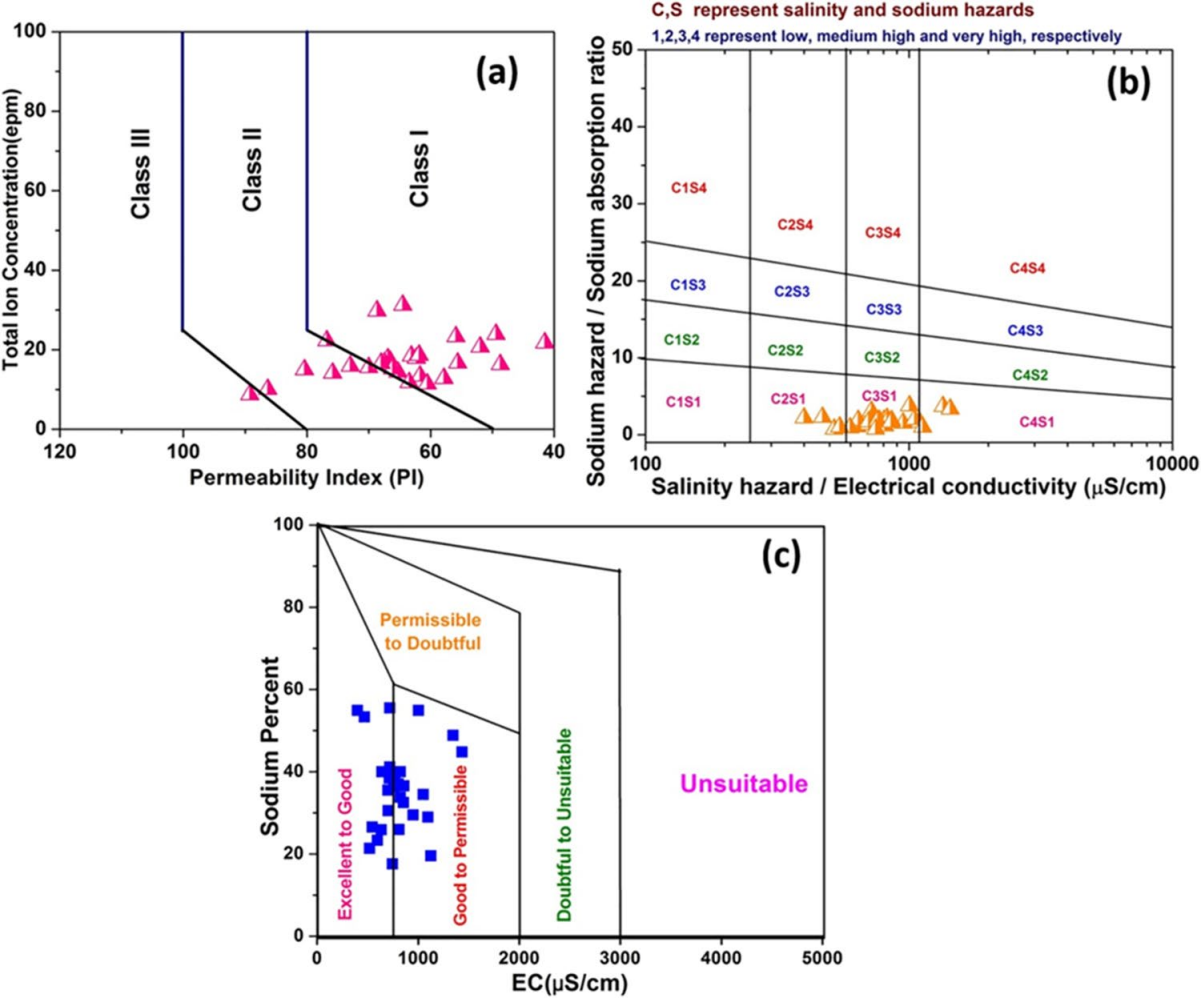
**a** Doneen’s diagram of water samples demonstrating impact on permeability of soils, **b** USSL diagram of water samples demonstrating presence of sodium and salinity hazard, and **c** Wilcox plot depicting irrigation suitability of water samples based on EC and Na%

Higher permeability of soil helps in growth of plants and crops. Ions present in water can modify the permeability of soils and the extent of this effect can be quantified using PI. The PI values of water samples range from 41.5 to 89.2 (mean value: 65). According to Doneen’s diagram, most of samples (61%) fall under class I, which means 100% of maximum permeability and suitability for irrigation. Few samples fall under class II with 75% of maximum permeability and only one sample falls in class III with 25% of maximum permeability (Fig. 5a).

Two different indicators are simultaneously plotted and studied for the associated hazards. The combined impact of SAR and EC is displayed by USSL diagram which is classified into 16 categories related to the SAR represented by S and EC represented by C. For each indicator, four different classes are defined on the basis of risk, i.e., low (1), medium (2), high (3), and very high (4), and overall risk is evaluated by combining individual risks (C1S1 (suitable) and C4S4 (unsuitable)). In the study area, the hazard trend is found to be C3S1 > C2S1 > C4S1 and most of the samples exhibit high salinity hazard and low sodium hazard (C3S1), which is followed by medium salinity hazard and low sodium hazard (C2S1 class). A few samples fall under C4S1 class representing very high salinity hazard and low sodium hazard. Based on USSL diagram, it can be suggested that most of the samples have high salinity hazard and low sodium hazard (Fig. 5b).

The combine effect of Na% and EC is depicted by Wilcox plot (Fig. 5c), which provides a better evaluation of suitability of water for irrigation. In this diagram, water quality is divided into five classes. Low Na% and low EC is considered as most suitable and high Na% and high EC is considered as most unsuitable. It can be inferred that all the water samples fall under excellent to permissible class and the water quality is suitable for irrigation but slow degradation and unsuitability of water quality is increasing with excessive flow of pollutants in the water system (Fig. 5c). Based on the USSL and Wilcox diagrams, it can be inferred that though salinity hazard is relatively higher, the sodium hazard is lower, suggesting presence of other salts which can be attributed to urbanization. Crop rotation, artificial recharge and conjunctive water use would be helpful to achieve sustainable water management.

### Assessment of water quality for drinking

#### Suitability based on guideline values

TDS and TH are the traditionally used indicators for assessing the water suitability for drinking. High TDS indicates the presence of more dissolved salts originating from weathered rocks or anthropogenic activities. Consumption of high TDS water may result into laxative and constipation in human (WHO 2011). Based on the TDS, water quality has been classified into four classes (Davies and DeWiest 1966), viz., desirable for drinking (TDS < 500 mg/L), permissible for drinking (TDS 500–1000 mg/L), useful for irrigation (TDS 1000–3000 mg/L) and unfit for both irrigation and drinking (TDS > 3000 mg/L). It is observed that all the samples fall either in desirable (43%) or permissible (57%) classes (Table 5). The desirable class indicates the cleanest and safest water, while permissible class indicates the alternate water to be used in case of zero availability of clean water. The water quality in the remaining two classes is not suitable for direct drinking purpose.

**Table 5:** Classification of drinking suitability of water based on TDS and TH

Water quality	Ranges	% distribution
TDS (mg/L)		
Desirable for drinking	< 500	43
Permissible for drinking	500–1000	57
Useful for irrigation	1000–3000	0
Unfit for drinking and irrigation	> 3000	0
TH (mg/L)		
Soft	< 60	0
Moderately hard	60–120	7
Hard	121–180	4
Very Hard	> 181	89

TH is another important indicator to check the potability of water. The dissolved salts of Ca^2+^ and Mg^2+^ ions are responsible for the hardness in water. Direct ingestion of hard water is not good for health and may lead to calcification of arteries and kidney related diseases (Sengupta 2013). TH (hard to very hard) leads to formation of soap scum, clogged pipes, dry skin and hair, corrosion of appliances. TH of the water samples ranges from 100 to 460 mg/L (mean value: 281 mg/L) (Table S2). Based on BIS (Bureau of Indian Standards) (2012) limits, 89% of the samples fall between desirable limit (200 mg/L) and maximum permissible limit (600 mg/L) (Table 3). Durfor and Becker (1964) classified the water samples into four classes based on TH, viz., soft (< 60 mg/L), moderately hard (60–120 mg/L), hard (121–181 mg/L), and very hard (> 181 mg/L). About 89% of the water samples fall in the very hard class whereas 7% and 4% of samples fall under moderately hard and hard classes respectively (Table 5). Soft water is safe for drinking, whereas, moderately hard water can be used after boiling. Hard and very hard water samples are consisted of permanent hardness and not qualified for the drinking. Compared to surface waters all shallow and deep groundwater samples have high hardness and salinity. TH of all the samples including surface waters fall above the DL of BIS (Bureau of Indian Standards) (2012) (Table 3).

#### Water quality indices (WQIs)

**Fig. 6:**
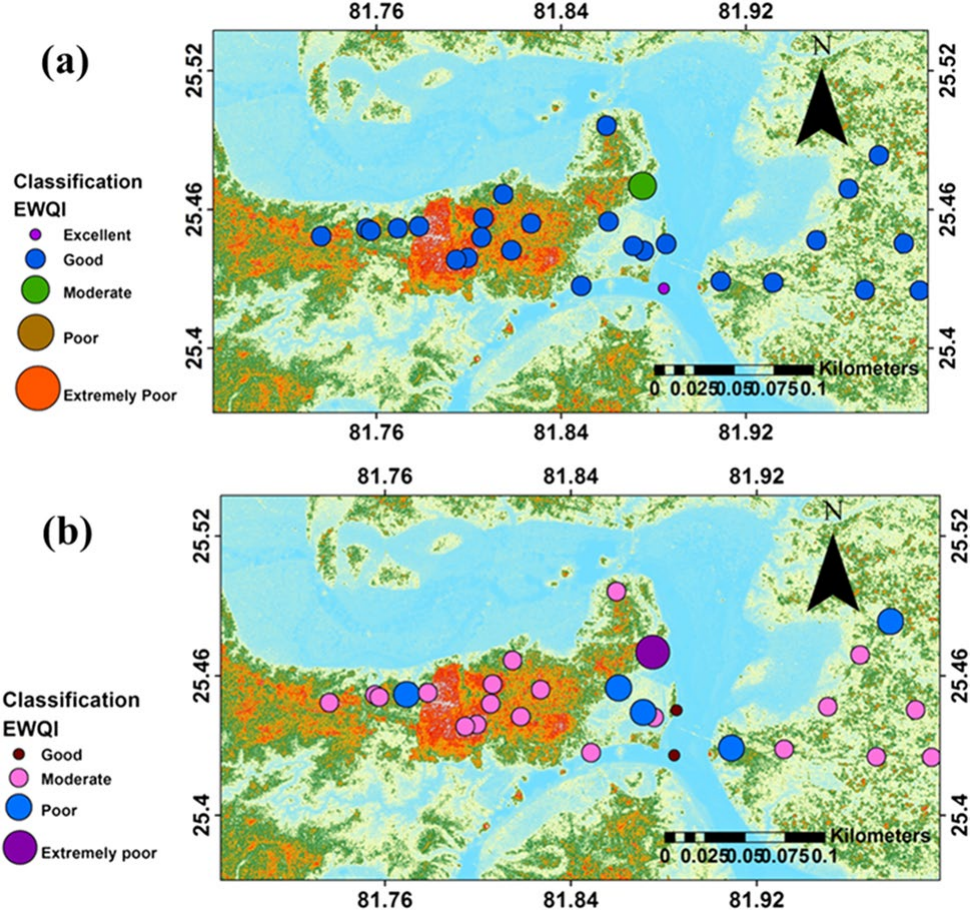
Bubble map representing the water quality based on EWQI classification using standards **a** WHO (2011) and **b** BIS (Bureau of Indian Standards) 2012

WQIs are valuable and efficient techniques to appraise the drinking and irrigation water quality of any area. Index calculation is the aggregation and conversion of all positive and negative footprints into single value, which is crucial for easy assessment of water quality. EWQI is one of the water quality probing indices, in which all the measured parameters of significance are aggregated into single index. In this study, all the total 28 water samples were examined for EWQI. The chosen parameters for assigning weights are TDS, pH, F^−^, Cl^−^, NO_3_^−^, SO_4_^2−^, Mg^2+^, Ca^2+^, Na^+^, K^+^, HCO_3_^−^, and TH. The relative weights for each parameter are given in supplementary table (Table S3), and EWQI was calculated using WHO (2011) and BIS (Bureau of Indian Standards) (2012) guideline values. Parameters like SO_4_^2−^, Cl^−^, and K^+^ are found to fall under critical category (*W*_*i*_ > 0.1) and remaining parameters (TDS, pH, F^−^, NO_3_^−^, Mg^2+^, Ca^2+^, Na^+^, and TH) under semi-critical category (*W*_*i*_: 0.05–0.1). It is observed that HCO_3_^−^falls under critical or semi-critical category. On the basis of criticality, the order of ions is found to be SO_4_^2−^ > Cl^−^ > K^+^ > pH > Na^+^ > Ca^2+^ > Mg^2+^ > F^−^ > NO_3_^−^ > TDS > TH. With reference to WHO standards, the EWQI values range from 25 to 52.5 (mean value 34.2) and only one sample falls under excellent (< 25) category. About 92% of the samples fall under the good (25–50) category and only one sample falls under moderate (51—75) category (Fig. 6a). Based on the BIS (Bureau of Indian Standards) (2012) standard, the EWQI values range from 39.8 to 108.7 (mean value 67.7) and 89% of the samples fall within moderate to poor category while 7% and 4% samples under good and very poor categories respectively (Table 6, Fig. 6b). The overall water quality of groundwater in the study area falls under moderate category, which suggests that there is an immediate need to improve the water quality situation so that future threat to water supplies can be avoided. Timely monitoring of water quality and mitigating the associated hazards through remedial measures is very essential in this region to achieve SDG-6 and 11. Sample-wise EWQI values are shown in Fig. 6.

**Table 6:** Percentage distribution of water quality of study area on the basis of EWQI

EWQI	Ranges	% distribution (WHO 2011)	% distribution BIS (Bureau of Indian Standards) (2012)
Excellent	< 25	4	0
Good	25–50	92	7
Moderate	51–75	4	71
Poor	76–100	0	18
Extremely Poor	> 100	0	4

**Fig. 7:**
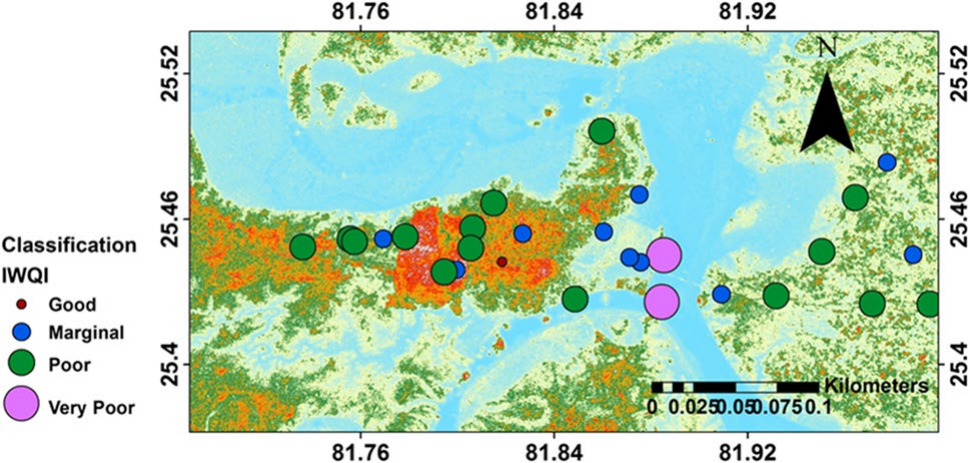
Bubble map representing the water quality based on IWQI classification

IWQI follows same principle of aggregation for all the parametric concentration into a single one as discussed for EWQI, but the steps applied for calculation are different. In this method, a new limit value is generated within the minimum and maximum acceptable guideline values (Mukate et al. 2019). For the calculation of IWQI parameters like, pH, TDS, HCO_3_^−^, Cl^−^, F^−^, NO_3_^−^, SO_4_^2−^, TH, Ca^2+^, and Mg^2+^ were considered. The parameters are weighted with subindex values based on BIS (Bureau of Indian Standards) (2012) guideline values for all the collected water samples. The estimated IWQI values of samples range from 1.9 to 5.23 (mean value: 3.28), and it is observed that most of the samples fall in either marginal (2–3) or poor (3–5) classes accounting for 35.7% and 53.6% of samples respectively (Table 7). Only 3.6% samples fall under good class (1–2) and 7.1% under unsuitable class (> 5) while none of the sample represent excellent class (< 1). The sample-wise IWQI values are shown in Fig. 7, which indicates that the unsuitable sample sites coincide with extremely poor sample sites based on EWQI values (Fig. 6).

**Table 7:** Percentage distribution of water quality of study area on the basis of IWQI

IWQI	Ranges	% distribution
Excellent	< 1	0
Good	1 to 2	4
Marginal	2 to 3	36
Poor	3 to 5	54
Unsuitable	> 5	7

#### Water pollution indices

Pollution Index is easy to handle and fast technique to extract the pollution causing factors from the selected chemical parameters. IWPI provides a clear indication of polluted water and the pollutant. IWPI was estimated for only chemical parameters having standard limit. Parameters used for the calculation of IWPI were pH, EC, TDS, HCO_3_^−^, Cl^−^, F^−^, NO_3_^−^, SO_4_^2−^, TH, Ca^2+^, and Mg^2+^. The IWPI values range from 0.44 to 1.41 (mean value: 0.85) and the major pollution contributors are in the order of EC > Mg^2+^ > TDS > TH > Ca^2+^ > HCO_3_^−^.

**Fig. 8:**
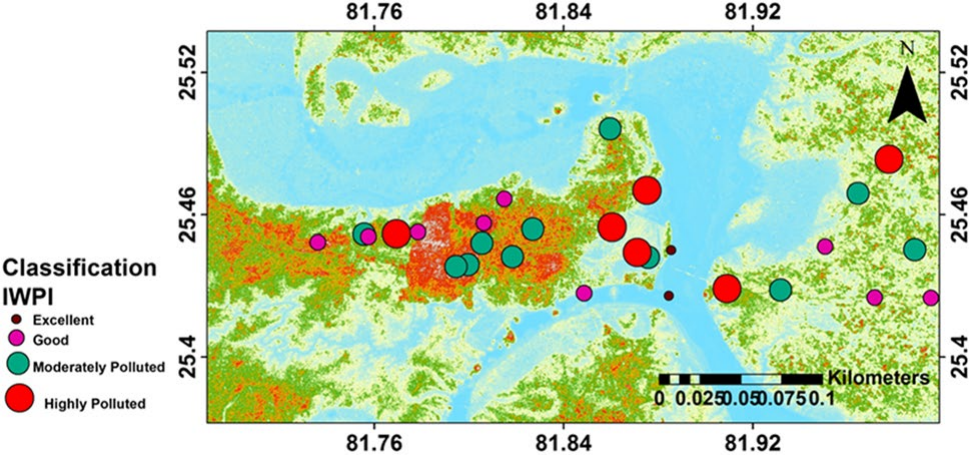
Bubble map representing the water quality based on IWPI classification

Based on IWPI values, most of the samples fall under moderately polluted (0.75–1) and highly polluted (> 1) water classes, accounting 39% and 21% of samples respectively. About, 7% and 32% of samples fall under excellent (< 0.5) and good (0.5–0.75) water classes respectively (Table 8). The sample-wise IWPI values are shown in Fig. 8, which indicates a wide contrast and high scatter in the water quality of this area. The major contributors for poor quality of groundwater are found to be TH, Mg^2+^, and TDS.

**Table 8:** Percentage distribution of water quality on the basis of IWPI

Water quality	Values	% distribution
Excellent	< 0.5	7
Good	0.5–0.75	32
Moderately polluted	0.75–1	39
highly polluted	> 1	21

It is found that 10 out of 28 samples indicate NO_3_^−^ concentration more than 10 mg/L and the highest concentration (> 45 mg/L) is found in shallow groundwater (S-25, 84 mg/L). Since the study area is urbanized and agriculturally active, it is imperative to mark the nitrate impacted water zones. The Nitrate Pollution Index (NPI) is a measure of potential nitrate pollution of water. It is a numerical index that is used to assess the risk of nitrate pollution in a given area based on factors such as soil type, climate, land use, and agricultural practices. NPI classifies water samples into 5 categories, viz., clean (NPI < 0), light (NPI 0–1), moderate (NPI 1–2), significant (NPI 2–3), and very significant (NPI > 3). Water samples with NPI values > 3 suggest unsuitability for both agriculture and drinking while samples with moderate to significant NPI values need to be treated before consumption, especially for women and children. Based on NPI, majority of the samples (57%) are found to be safe. About 14%, 11%, 14%, and 4% of samples fall under lightly, moderately, significantly, and very significantly polluted categories respectively (Table 9). It is found that only a small percentage (18%) of samples has the potential to affect human health in the study area.

**Table 9:** Percentage distribution of nitrate level of study area on the basis of NPI

Pollution level based on nitrate	Range	% distribution
No pollution	< 0	57
Light pollution	0–1	14
Moderate pollution	1–2	11
Significant pollution	2–3	14
Very significant pollution	> 3	4

#### Sensitivity analysis

**Fig. 9:**
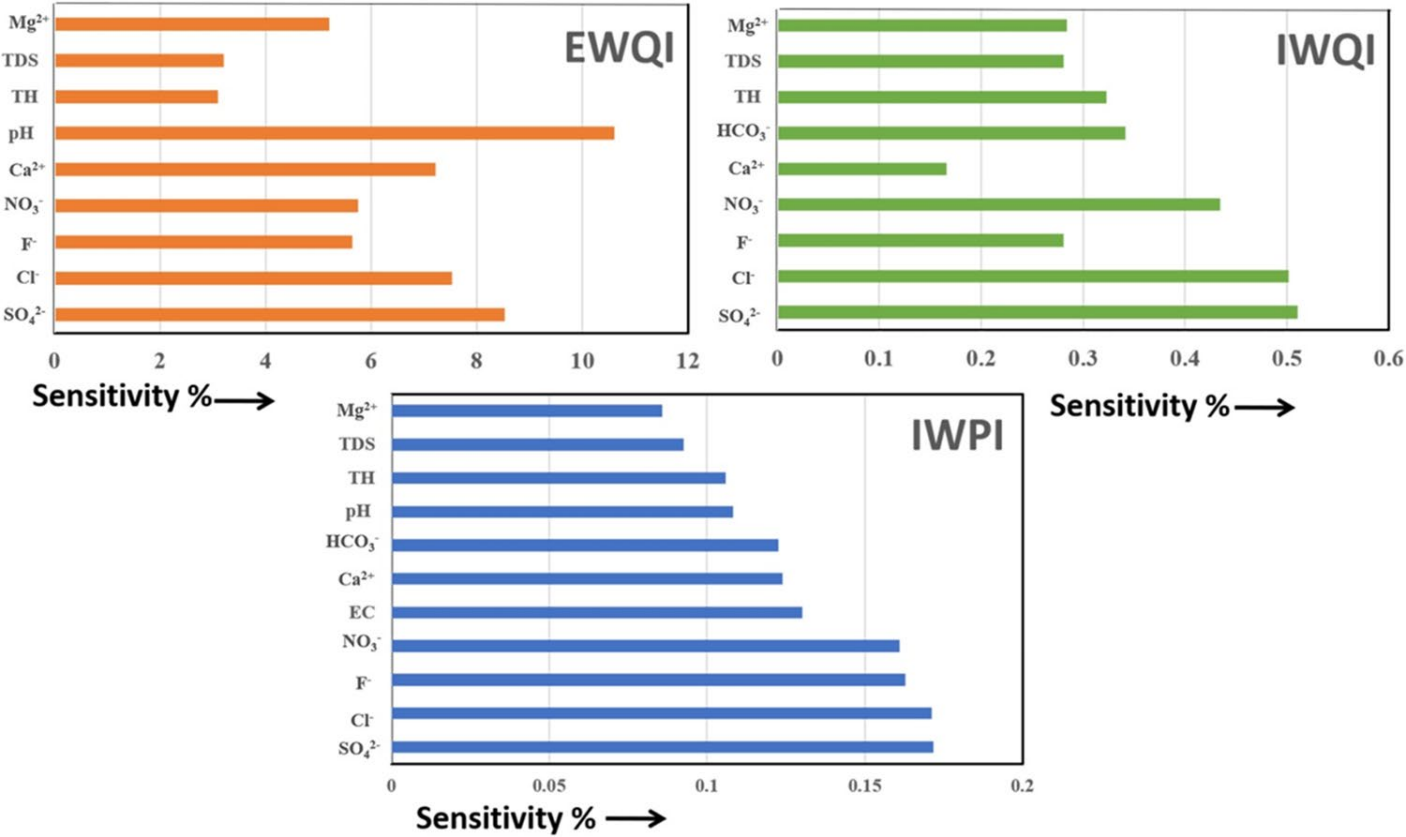
Sensitivity percentage for the EWQI, IWQI and IWPI

In the case of IWPI, removal of each input parameter results in sensitivity values ranging from 0.09% to 0.17%. The SO_4_^2− ^is found to be the most influential parameter impacting IWPI score, and value ranges from 0.06% to 0.84% (mean value: 0.17%). Very minor effect is noticed for Mg^2+^, with sensitivity values ranging from 0.007% to 0.76% (mean value: 0.09%). The order of influence of different parameters is as follows; SO_4_^2−^ > Cl^−^ > F^−^ > NO_3_^−^ > EC > Ca^2+^ > HCO_3_^−^ > pH > TH > TDS > Mg^2+^. The sensitivity of IWQI ranges from 0.17 to 0.51% and SO_4_^2−^is found to be most influential ion, with values ranging between 0.12% and 0.74% (mean value: 0.51%). Ca^2+^is the least influential parameter with values ranging between 0.001% and 0.47% (mean value: 0.17%). The order of influence is found to be; SO_4_^2−^ > Cl^−^ > NO_3_^−^ > HCO_3_^−^ > TH > Mg^2+^ > F^−^ > TDS > Ca^2+^. The average sensitivity values of EWQI is found to range from 3.09% to 10.6%. pH was the most influential parameter (0.44% to 17.3%, mean value: 10.6%) and TH the least (1.33% to 4.92%, mean value: 3.09%). The order of influence is found to be; pH > SO_4_^2−^ > Cl^−^ > Ca^2+^ > NO_3_^−^ > F^−^ > Mg^2+^ > TDS > TH (Fig. 9).

The sensitivity values depicted in Fig. 9 suggest that pH is the main influential factor in deciding the category of EWQI followed by the SO_4_^2−^ and Cl^−^ concentrations. Similar results were observed in IWQI except for pH, since it is not considered as input parameter. IWPI also shows SO_4_^2−^ and Cl^−^ ions as the most sensitive parameters, which suggests that anthropogenic inputs exert a greater control on the water quality than natural factors. Figure 9 also indicates that F^−^ ion is not a major sensitive parameter in the case of EWQI and IWQI while IWPI exhibits a significant sensitivity towards F^−^. However, considering the health hazard due to consumption of high F^−^ waters, all the WQIs should have shown higher sensitivity towards F^−^. This apparent contradiction demonstrates that conventional index systems are limited by subjective weightage of ions and bias towards the higher permissible limit values. Therefore, a more robust approach of water quality indexing, such as fuzzy logic approach, is necessary.

**Fig. 10:**
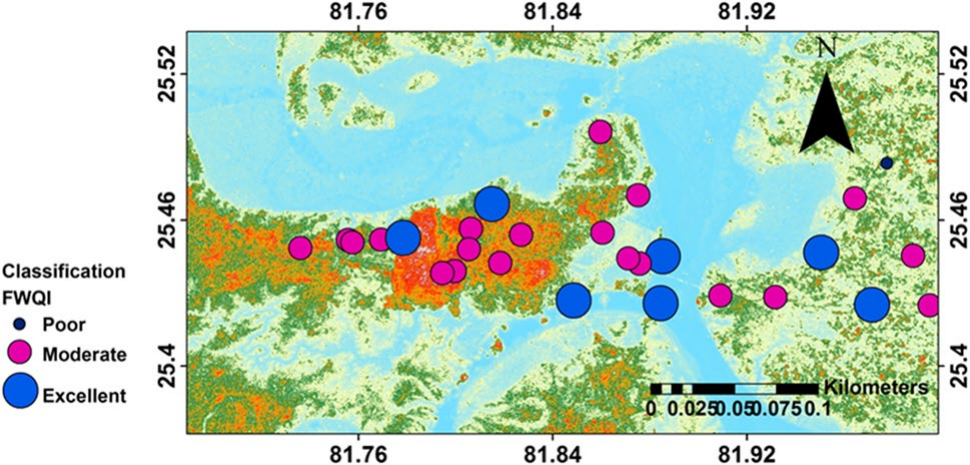
Bubble map representing the water quality based on FWQI classification

#### Fuzzy water quality index (FWQI)

The fuzzy rules were formulated based on the triangular membership functions giving higher weightage to important water quality parameters in the study area, viz., NO_3_^−^, F^−^, TDS, and TH (Tables 1 and 2). The fuzzy output was obtained from the Mamdani FIS algorithm using MATLAB v.2016a. The output obtained from each rule is a resultant of respective output membership functions and ‘min’ implication method (AND operator) of the FIS. These outputs are then combined into a single fuzzy set based on the “max” (OR operator) aggregation method. To compute a final crisp output value, the resultant combined output fuzzy set is defuzzified based on “centroid” defuzzification method. The FWQI values range from 43.9 to 87.5 (mean value: 62.2), and majority of the samples fall under moderate (71%), which is similar to other WQIs. However, a relatively higher number of samples fall in excellent category (25%) in the case of FWQI as compared to other WQIs (Table 10), which can be attributed to removal of subjective weightage of ions based on MPL values of BIS 2012 and assigning higher weightage for NO_3_^−^, F^−^, TDS, and TH. The sample-wise FWQI values are plotted in the Fig. 10, which indicate that water quality is moderate to poor across the study area.

**Table 10:** Percentage distribution of water quality of study area on the basis of FWQI

FWQI	Ranges	% distribution
Excellent	75–100	25
Moderate	45–75	71
Poor	< 45	4

## Discussion

This section provides a comprehensive analysis of the results obtained in this study with the current water quality status in MGP and its implication on future sustainability of water resources in Prayagraj area. We have also conducted a thorough comparative analysis of various WQIs obtained in this area and commented their relevance to other highly urbanized and irrigated regions. Variation in WQIs with respect to source water was evaluated to deduce possible geochemical reactions controlling water quality in the study area.

The chemical quality of the water samples in the study area clearly suggests that most of the of samples are suitable for irrigation while more than half of the samples do not conform to the drinking water limits, due to presence of higher TDS, TH and Mg^2+^. The average TDS and TH values (542 mg/L and 281 mg/L, respectively) obtained in this study are found to be similar in other parts of Ganga basin as reported by Hasan and Rai (2020). The seasonal variation of TDS values of groundwater suggests that premonsoon water quality is poorer compared to postmonsoon, which could be due to dilution by rainwater infiltration (Kumar et al 2022). This supports the earlier argument that impact of urbanization would be more intense on water resources during premonsoon season (“Water sample collection” section). Contribution of anthropogenic factors towards degradation of water resources in MGP has been documented by several researchers (Jaiswal and Pandey 2021; Soni et al. 2022). Water quality has been found to be suitable for irrigation across Prayagraj district under this study, as well as in other regions of MGP (Bhatt et al. 2021). Only in a few instances, the suitability was impacted due to presence of high MH and RSC values (Table 4). Presence of high MH as reason for unsuitability of water for irrigation is also reported by Maurya and Saxena (2022) in MGP. This study also reported low sodium hazard, which is also observed in other parts of the MGP (Kumar et al 2022). The redox condition of the aquifers in Ganga basin has been studied, and it is reported that aquifer redox chemistry is mainly oxic to slightly anoxic in nature (Mukherjee 2018). This implies possibility of release of redox sensitive metals in to groundwater, which would further hamper the water quality. From the above discussion it can be comprehended that water resources might be more stressed towards drinking water supplies as compared to irrigation needs, considering the quality. This might adversely impact the “clean water and sanitation” goal as advocated under SDG-6. Control of dumping of untreated urban wastes as well as increasing the groundwater levels through artificial recharge measures can be instrumental in achieving the SDG-6 and 11.

Unlike, single parametric assessment, index-based approach is an efficient method for providing the overall groundwater quality. This is achieved by calculating EWQI, IWQI, IWPI, and FWQI in this study. The EWQI and IWQI values suggest that majority of the samples can be classified as “moderate,” and similar findings were reported by Hasan and Rai (2020) and Gautam et al (2022) in Prayagraj district and other parts of MGP. Very few studies have attempted IWPI to evaluate the water quality in MGP. The IWPI values obtained in this study signify that water samples are moderately polluted. A comparison of the IWPI values with the results of recent study by Maurya and Saxena (2022), clearly suggests that the water quality has been degrading with time and urbanization has been a major contributor. This is further supported by sensitivity analysis, which indicates SO_4_^2− ^followed by Cl^−^ as the major influencing ions controlling WQIs in this area (Section 4.5). NPI values also indicate that water samples are moderately contaminated, which is well supported by studies conducted in floodplains of Ganga (Madhav et al. 2018). Prevalence of high NO_3_^−^ in groundwaters of Lower Ganga Plains has been reported by several researchers (Mukherjee and Singh 2021; Shukla and Saxena 2020; Nijesh et al. 2021).

**Fig. 11:**
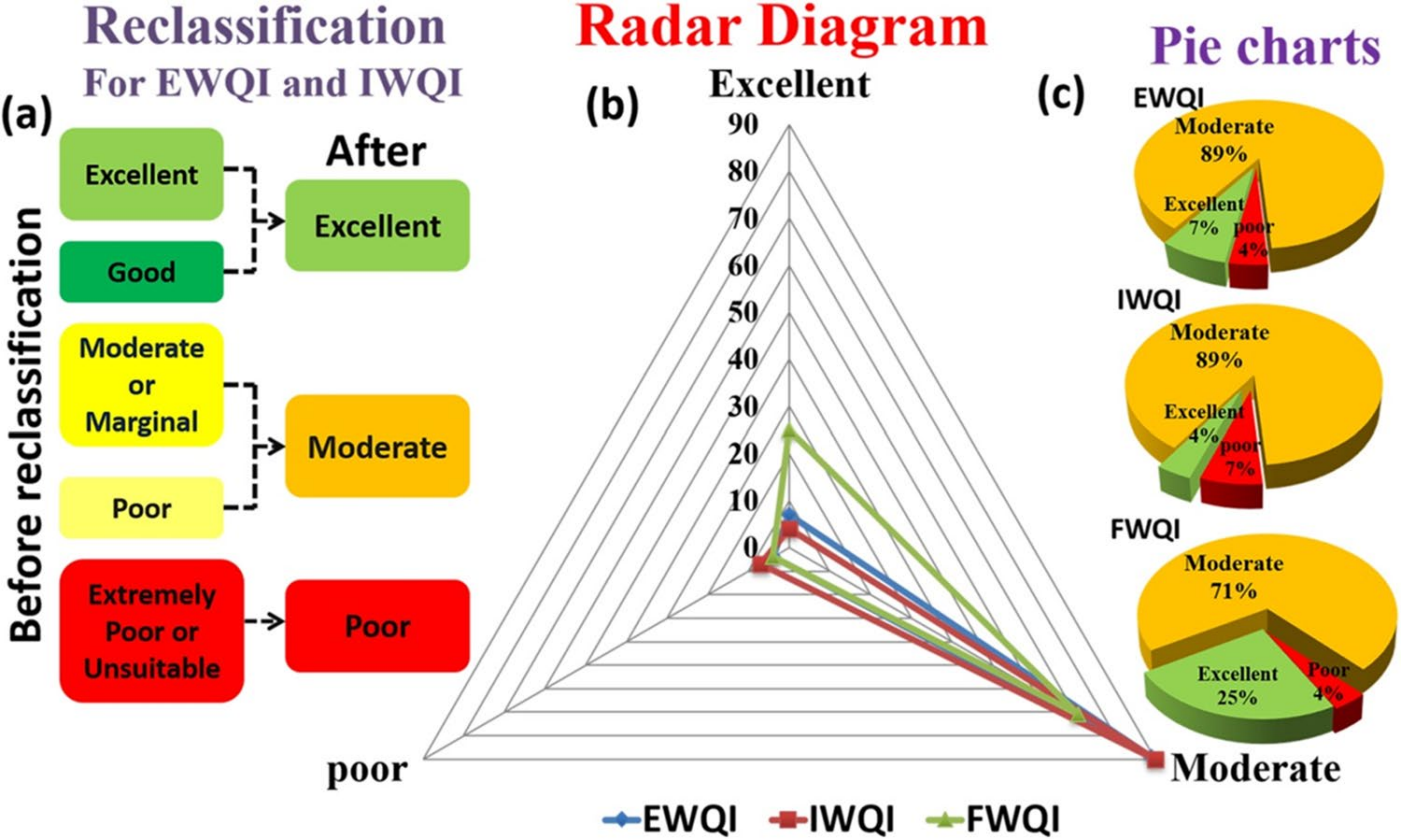
**a** Reclassification method for EWQI and IWQI, **b** radar diagram depicting the comparison of IWQI, EWQI and FWQI outputs, and **c** pie charts depicting the percentage distribution of water quality based upon EWQI, IWQI, and FWQI

The above indices are not user-defined and therefore do not allow prioritization of the contaminants based on the area of interest and user requirement. In addition, the conventional WQIs are constrained by BIS and WHO limits and therefore manipulation of weighting factors based on field understanding and human experience is not feasible. In this context, FWQI is an apt method as it allows manual weight assignment to globally critical parameters, like, NO_3_^−^, F^−^, TDS, and TH. There are five categories in EWQI and IWQI (Tables 6 and 7) while FWQI displays three types of water quality (Table 10), therefore in order to compare these indices, EWQI and IWQI were reclassified into three groups. The reclassification is done by merging categories as shown in Fig. 11a. The samples falling in “poor” class in EWQI and IWQI were merged with “Moderate” class, since the values were more towards the “Moderate” class values. For all indices, BIS (Bureau of Indian Standards) (2012) standard was considered specifically for the reclassification. The excellent quality indicates that the water quality is free from threats and contaminants are within natural levels. Moderate quality indicates that water quality is close to contaminant threat level and slightly deviated from the its natural level. Poor quality resembles high risk of pollution and significant diversion from the natural level. The comparison of EWQI, IWQI, and FWQI outputs is shown using Radar diagram (Fig. 11b). Figure shows that 89% of water samples under moderate category in the case of EWQI and IWQI as compared to 71% in the case of FWQI (Fig. 11c). However, a higher percentage of samples falls in excellent category as per FWQI (25%) as compared to EWQI and IWQI (4–7%). Studies have reported that based on FWQI values the water quality can be classified as “poor” in Ganga basin (Srinivas and Singh 2018; Shukla 2020). A similar finding was reported by Singh et al. (2021), where fuzzy modeling of the water chemistry data during 1990–2016 indicated that the water quality of Ganga basin was poor to unsuitable. Fuzzy logic method has been found to be very useful in dealing with the ambiguity in environmental issues, non-linearity, and uncertainty in the input data. These features in the fuzzy logic are essentially derived from its capacity to apply linguistic terms that is impossible for other WQI (Semiromi et al. 2011; Chaudhary 2020; Abidi et al. 2024). Based on the observations from this study and reported studies, FWQI can be regarded as a better indicator in assessing the water quality in urbanized areas.

**Fig. 12:**
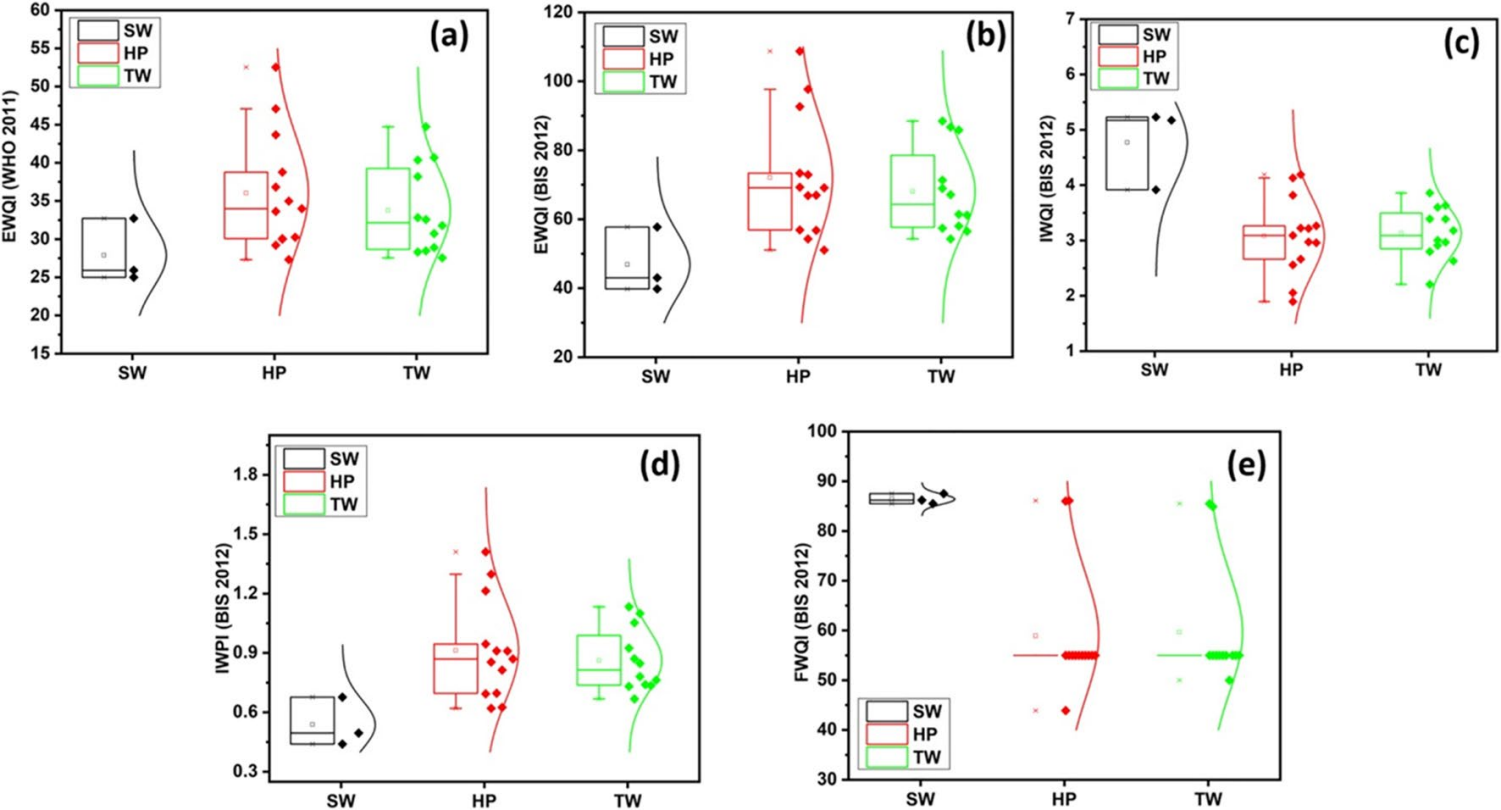
Box whisker plots representing water quality range from different sources based on **a** EWQI (WHO 2011), **b** EWQI (BIS (Bureau of Indian Standards) 2012), **c** IWQI (BIS (Bureau of Indian Standards) 2012), **d** IWPI (BIS (Bureau of Indian Standards) 2012), and **e** FWQI (BIS (Bureau of Indian Standards) 2012)

Table 11 presents the WQIs statistics of water samples based on the sources. Surface waters (3 nos) showed that EWQI (WHO 2011) and FWQI fall under good category while IWQI and IWPI suggest moderate pollution. The EWQI and FWQI of shallow groundwater (13 nos) indicate that majority of the samples fall under good category (> 77%) except in the case of IWQI and IWPI, which showed more samples under moderately polluted category. Deep groundwater also demonstrated a similar pattern of WQIs as that of shallow groundwater. Figure 12 depicts the spread in the WQIs data in various sources. It is clear from the Fig. 12 that both shallow and deep groundwater samples exhibit similar spread in WQIs with some high values in the case of shallow groundwater samples.

**Table 11:** Percentage distribution of water quality of study area on the basis of all indices and different water sources

Indices	Classes	SW (3) (%)	HP (13) (%)	TW (12) (%)
EWQI (WHO 2011)	Excellent	–	–	–
	Good	100	92	100
	Moderate	–	8	–
	Poor	–	–	–
	Extremely poor	–	–	–
EWQI (BIS (Bureau of Indian Standards) 2012)	Excellent	–	–	–
	Good	67	–	
	Moderate	33	92	100
	Poor	–	8	–
	Extremely poor	–	–	–
IWQI (BIS (Bureau of Indian Standards) 2012)	Excellent	–	–	–
	Good	–	8	–
	Marginal	–	38	42
	Poor	33	54	58
	Unsuitable	67	–	–
IWPI (BIS (Bureau of Indian Standards) 2012)	Excellent	–	–	–
	Good	–	31	33
	Moderately polluted	33	46	42
	Highly polluted	67	23	25
FWQI (BIS (Bureau of Indian Standards) 2012)	Excellent	100	8	–
	Moderate	–	77	77
	Poor	–	15	23

Factors responsible for contamination in water are mainly contributed from minerals present in the formation or anthropogenic sources. Most of the shallow and deep aquifer samples showed moderate to high TH suggesting contribution of Ca^2+^ and Mg^2+^salts. Similarly, fluoride can be contributed by various clay minerals present in the formation. However, nitrate sources are mainly anthropogenic. Geology of the subsurface indicates that the water bearing formations are mainly sandy and the major minerals are quartz, clay, feldspar with occasional limestone and shale. These features are common for both shallow and deep aquifers. Therefore, based on similar aquifer chemistry and geology, the following geochemical reactions can be deduced.

21 $${\mathrm{2KAlSi}}_{3}{\mathrm{O}}_{8}\mathrm{(K-feldspar) +2H}^\mathrm{+}+ \mathrm{9H}_{2}{\mathrm{O}}\to {\mathrm{Al}}_{2}{\mathrm{Si}}_{2}{\mathrm{O}}_{5}\left({\mathrm{OH}}\right)_{4}\mathrm{ (Kaolinite)+2K}^\mathrm{+}+ \mathrm{4H}_{4}{\mathrm{SiO}}_{4}$$

22 $${\mathrm{2NaAlSi}}_{3}{\mathrm{O}}_{8}\text{(Albite) +H}^\mathrm{+}+ \mathrm{7H}_{2}{\mathrm{O}}\to {\mathrm{Al}}\left({\mathrm{OH}}\right)_{3}\mathrm{ (Gibbsite)+Na}^\mathrm{+}+ \mathrm{3H}_{4}{\mathrm{SiO}}_{4}$$

23 $${\mathrm{CaA}1}_{2}{\mathrm{Si}}_{2}{\mathrm{O}}_{8}\left(\mathrm{Anorthite}\right)+{2\mathrm{H}}^{+}+{6\mathrm{H}}_{2}\mathrm{O}\to 2\mathrm{A}1{\mathrm{OH}}_{3}\left(\mathrm{Gibbsite}\right)+{\mathrm{Ca}}^{2+}+{2\mathrm{H}}_{4}{\mathrm{SiO}}_{4}$$

24 $${\mathrm{2NaAlSi}}_{3}{\mathrm{O}}_\mathrm{8}\mathrm{(Albite) + 2CO}_{2}\mathrm{ + 6H}_{2}{\mathrm{O}}\to {\mathrm{Al}}_{2}{\mathrm{Si}}_{4}{\mathrm{O}}_{10}\left({\mathrm{OH}}\right)_{2}\text{(Montmorillonite) + 2Na}^+ \mathrm{ } + \mathrm{ HCO}_{3}-\mathrm{ + 2H}_{4}{\mathrm{SiO}}_{4}$$

25 $${\mathrm{CaCO}}_{3}\left({\mathrm{Calcite}}\right)+ \mathrm{H}_{2}{\mathrm{CO}}_{3}\to {\mathrm{Ca}}_\mathrm{2} + + \mathrm{2HCO}_{3}^{-}$$

26 $${\mathrm{KAl}}_3{\mathrm{Si}}_3{\mathrm O}_{10}{\left(\mathrm{OH},\;\mathrm F\right)}_2\left(\mathrm{Muscvite}\right)+{\mathrm{CO}}_2+2.5{\mathrm H}_2\mathrm O\rightarrow1.5{\mathrm{Al}}_2{\mathrm{Si}}_2{\mathrm O}_5{\left(\mathrm{OH}\right)}_4\left(\mathrm{Kaolinite}\right)+\mathrm K^++2\mathrm F^-+\mathrm{HCO}_3^-$$

## Conclusion

An integrated and systematic approach is applied to critically evaluate the water quality of a highly urbanized and irrigated region of MGP, viz., Prayagraj district of Uttar Pradesh, India. The water quality demonstrates a spatial variation ranging from excellent to very poor category. TDS and TH are found to be higher while F^−^ and NO_3_^−^ are found to be mostly safe towards drinking. EWQI and IWQI demonstrate good to moderate water quality for majority of the samples while pollution indices (IWPI and NPI) classified waters into moderately to highly polluted category. Sensitivity analysis of these different indices shows that SO_4_^2−^, Cl^−^, F^−^, NO_3_^−^ and EC are the most influential parameters. Fuzzy Water Quality Index was calculated prioritizing TDS, TH, F^−^, and NO_3_^−^ and the values suggest moderate quality of water. A comprehensive analysis of different WQIs adopted in this study was undertaken by reclassifying the categories. It is found that FWQI provides a better representation of water quality providing suitable weighting factors to influential ions based on the study area. The spread in the WQI data in different sources of water suggests that shallow and deep groundwater quality is similar but different from surface water. Combining the geological and WQI information, possible geochemical reactions contributing to water chemistry are deduced. This study finds that the water quality towards drinking purposes has been declining considerably and an immediate planning is needed to check the water quality degradation. The outcome from this study is not only useful to Prayagraj district but also relevant to similar urban regions of Ganga basin.

## Supplementary Information

Below is the link to the electronic supplementary material.

Supplementary file1 (DOCX 43 KB)

## Data Availability

The authors declare that the data supporting the findings of this study are available within the paper as Supplementary Information.
