# Supplementary material for: Comprehensive assessment of groundwater quality in the Prayagraj District, Ganga Basin
Source: Environ Sci Pollut Res Int. 2024 Jul 9;32(6):3238–60. doi: 10.1007/s11356-024-34030-1 (PMC11832705; doi:10.1007/s11356-024-34030-1)
Supplement: Supplementary file 1 — Supplementary file1 (DOCX 43 KB) [file 11356_2024_34030_MOESM1_ESM.docx]

# Supplementary Information

**Comprehensive Assessment of Groundwater Quality in the Prayagraj District, Ganga Basin**

Bhumika Kumari^1,2^, Tirumalesh Keesari^1,2*^, Annadasankar Roy^1,2^, Hemant Mohokar^1^, Harish Jagat Pant^1^

*^1^Isotope Hydrology Section, Isotope and Radiation Application Division, Bhabha Atomic Research Centre, Mumbai 400 085, India*

*^2^Homi Bhabha National Institute, Mumbai 400 094, India*

*Contact details of the corresponding author:

Dr. Tirumalesh Keesari

Scientific Officer, Bhabha Atomic Research Centre

and Associate Professor, Homi Bhabha National Institute

Mumbai, India 400094

Email: [tiruh2o@gmail.com](mailto:tiruh2o@gmail.com); [tirumal@barc.gov.in](mailto:tirumal@barc.gov.in)

**Supplementary Tables:**

Table S1 Equations for calculating the water suitability indicators

Table S2 Measured values of hydrochemical parameters and their calculated averages

Table S3 Assigned relative weights for each parameter using EWQI

Table S4 Percentage distribution of water quality of study area on the basis of all indices and different water sources.

List of references

Table S1 Equations for calculating the water suitability indicators

| Assessment | Indicators and equations | References |
| --- | --- | --- |
| Suitability for drinking purposes | $TDS(mg/L)=0.64\times EC (\mu s/cm)$ | Hem 1985 |
|  | $TH (mg/L)=2.497\times{Ca}^{2+}+ 4.115 \times{Mg}^{2+}$ | Todd 1980 |
| Suitability for Irrigation | $SAR=\frac{{Na}^{+}}{\sqrt{\frac{{Ca}^{2+}+{Mg}^{2+}}{2}}}$ | Richards 1954 |
|  | $RSC(meq/L)=\left( {HCO}_{3}^{-}+{CO}_{3}^{2-} \right)-({Ca}^{2+}+{Mg}^{2+})$ | Eaton 1950 |
|  | $Na\%=\frac{({Na}^{+}+K^{+})\times100}{{Ca}^{2+}+{Mg}^{2+}+{Na}^{+}+K^{+}}$ | Wilcox 1948 |
|  | $MH= \frac{{Mg}^{2+}}{{Ca}^{2+}+ {Mg}^{2+}}\times100$ | Raghunath 1987 |
|  | $CR= \frac{({Cl}/{35.5)}+2 ({{SO}_{4}}/{96)}}{2\{({{CO}_{3}+ {HCO}_{3})}/{100\}}}$ | Balasubramanian 1986 |
|  | $PI=\frac{{Na}^{+}+\sqrt{{HCO}_{3}^{-}}}{{Ca}^{2+}+{Mg}^{2+}+{Na}^{+}}\times100$ | Doneen 1964 |

Table S2 Measured values of hydrochemical parameters and their calculated averages

| Sources | Sample-ID | pH | Temperature | EC | TDS | Ca^2+^ | Mg^2+^ | Na^+^ | K^+^ | F^-^ | NO_3_^-^ | Cl^-^ | SO_4_^2-^ | HCO_3_^-^ | TH |
| --- | --- | --- | --- | --- | --- | --- | --- | --- | --- | --- | --- | --- | --- | --- | --- |
| SW | S1 | 7.8 | 31.99 | 400 | 268 | 24.0 | 9.7 | 50.6 | 9.09 | 0.44 | 7.0 | 42.6 | 32.9 | 146 | 100 |
| SW | S2 | 7.9 | 32 | 720 | 482 | 32.1 | 21.9 | 92.3 | 9.10 | 0.39 | 4.5 | 92.2 | 46.1 | 232 | 170 |
| SW | S3 | 7.8 | 32 | 470 | 315 | 28.1 | 12.2 | 57.5 | 9.44 | 0.34 | 9.8 | 42.6 | 35.0 | 183 | 120 |
| DTW | S4 | 7.5 | 34.1 | 1004 | 673 | 36.1 | 38.9 | 138 | 3.69 | 0.39 | 0.7 | 99.3 | 115.3 | 366 | 250 |
| HP | S5 | 7.8 | 28.83 | 1347 | 902 | 88.2 | 38.9 | 165.6 | 2.01 | 0.21 | 2.5 | 127.7 | 122.3 | 537 | 380 |
| DTW | S6 | 7.6 | 31.2 | 1098 | 736 | 84.2 | 53.5 | 78.2 | 4.09 | 0.30 | 3.3 | 92.2 | 135.2 | 390 | 430 |
| DTW | S7 | 7.9 | 28.26 | 950 | 637 | 56.1 | 55.9 | 69.0 | 3.96 | 0.30 | 31.0 | 106.4 | 67.2 | 354 | 370 |
| DTW | S8 | 8 | 28.85 | 804 | 539 | 52.1 | 35.3 | 72.25 | 3.56 | 0.46 | 30.7 | 35.5 | 26.0 | 415 | 275 |
| DTW | S9 | 7.8 | 28.6 | 810 | 543 | 64.1 | 36.5 | 47.64 | 3.90 | 0.39 | 6.5 | 49.6 | 25.7 | 390 | 310 |
| HP | S10 | 7.9 | 29.1 | 635 | 425 | 48.1 | 31.6 | 38.28 | 3.18 | 0.40 | 4.1 | 21.3 | 4.1 | 366 | 250 |
| DTW | S11 | 7.8 | 30.87 | 704 | 472 | 48.1 | 31.6 | 48.73 | 3.24 | 0.43 | 5.0 | 21.3 | 8.4 | 390 | 250 |
| HP | S12 | 7.7 | 32.1 | 860 | 576 | 52.1 | 41.3 | 73.6 | 9.99 | 0.73 | 31.8 | 56.7 | 29.1 | 415 | 300 |
| HP | S13 | 8 | 28.31 | 825 | 553 | 56.1 | 31.6 | 81.1 | 2.77 | 1.00 | 0.8 | 70.9 | 36.9 | 366 | 270 |
| DTW | S14 | 7.9 | 34.15 | 642 | 430 | 40.1 | 26.8 | 62.62 | 3.07 | 0.50 | 1.4 | 14.2 | 16.0 | 390 | 210 |
| DTW | S15 | 7.8 | 29.07 | 772 | 517 | 56.1 | 29.2 | 70.61 | 3.57 | 0.24 | 7.4 | 42.6 | 41.4 | 384 | 260 |
| DTW | S16 | 7.8 | 30 | 720 | 482 | 28.1 | 40.1 | 73.6 | 3.28 | 0.35 | 3.0 | 21.3 | 35.3 | 403 | 235 |
| DTW | S17 | 7.7 | 28.57 | 716 | 480 | 40.1 | 34.0 | 66.82 | 3.42 | 0.35 | 4.0 | 35.5 | 28.3 | 378 | 240 |
| HP | S18 | 7.9 | 29.22 | 1435 | 961 | 72.1 | 63.2 | 161 | 5.33 | 0.54 | 35.7 | 99.3 | 111 | 622 | 440 |
| DTW | S19 | 7.8 | 31.4 | 703 | 471 | 52.1 | 29.2 | 61.18 | 3.75 | 0.43 | 17.4 | 35.5 | 32.6 | 354 | 250 |
| HP | S20 | 7.6 | 29.46 | 825 | 553 | 56.1 | 38.9 | 69 | 2.44 | 0.72 | 24.7 | 49.6 | 32.1 | 415 | 300 |
| DTW | S21 | 7.6 | 28.8 | 1050 | 704 | 56.1 | 58.4 | 89.73 | 3.67 | 0.54 | 10.4 | 113.5 | 102 | 390 | 380 |
| HP | S22 | 8 | 28.4 | 520 | 348 | 44.1 | 28.0 | 25.93 | 3.71 | 0.44 | 7.8 | 14.2 | 5.6 | 317 | 225 |
| HP | S23 | 7.8 | 28.05 | 598 | 401 | 64.1 | 21.9 | 33.45 | 2.55 | 0.35 | 21.7 | 28.4 | 13.9 | 317 | 250 |
| HP | S24 | 7.9 | 28.03 | 760 | 509 | 84.2 | 13.4 | 69.0 | 1.78 | 1.10 | 9.9 | 35.5 | 13.1 | 427 | 265 |
| HP | S25 | 7.5 | 28.37 | 1127 | 755 | 96.2 | 53.5 | 49.24 | 3.86 | 0.32 | 84.1 | 92.2 | 54.0 | 403 | 460 |
| HP | S26 | 7.6 | 28.6 | 855 | 573 | 56.1 | 43.8 | 68.26 | 4.41 | 0.59 | 12.9 | 35.5 | 7.7 | 488 | 320 |
| HP | S27 | 7.8 | 28.56 | 546 | 366 | 60.1 | 17.0 | 35.03 | 2.65 | 0.31 | 16.1 | 21.3 | 9.2 | 305 | 220 |
| HP | S28 | 7.8 | 28.9 | 747 | 500 | 76.2 | 36.5 | 31.69 | 2.78 | 0.46 | 21.1 | 42.6 | 2.2 | 415 | 340 |

Table S3 Assigned relative weights for each parameter using EWQI

| Parameters | Relative weights (W_i_) |
| --- | --- |
| TDS | 0.069118 |
| pH | 0.084956 |
| F^-^ | 0.076505 |
| Cl^-^ | 0.117807 |
| NO_3_^-^ | 0.070498 |
| SO_4_^2-^ | 0.119772 |
| HCO_3_^-^ | 0.045027 |
| Na^+^ | 0.083069 |
| K^+^ | 0.105644 |
| Mg^2+^ | 0.079212 |
| Ca^2+^ | 0.082003 |
| TH | 0.066388 |
| ∑W_i_ | 1 |

Table S4 Percentage distribution of water quality of study area on the basis of all indices and different water sources.

| Indices | Classes | SW (3) (%) | HP (13) (%) | TW (12) (%) |
| --- | --- | --- | --- | --- |
| EWQI (WHO 2011) | Excellent | - | - | - |
|  | Good | 100 | 92 | 100 |
|  | Moderate | - | 8 | - |
|  | Poor | - | - | - |
|  | Extremely Poor | - | - | - |
| EWQI (BIS 2012) | Excellent | - | - | - |
|  | Good | 67 | - |  |
|  | Moderate | 33 | 92 | 100 |
|  | Poor | - | 8 | - |
|  | Extremely Poor | - | - | - |
| IWQI (BIS 2012) | Excellent | - | - | - |
|  | Good | - | 8 | - |
|  | Marginal | - | 38 | 42 |
|  | poor | 33 | 54 | 58 |
|  | unsuitable | 67 | - | - |
| IWPI (BIS 2012) | Excellent | - | - | - |
|  | Good | - | 31 | 33 |
|  | Moderately polluted | 33 | 46 | 42 |
|  | highly polluted | 67 | 23 | 25 |
| FWQI (BIS 2012) | Excellent | 100 | 8 | - |
|  | Moderate | - | 77 | 77 |
|  | Poor | - | 15 | 23 |

**References:**

1. Balasubramanian A (1986) Hydrogeological investigations in the Tambraparani river basin, Tamil nadu. Unpublished PH.D. Thesis, University of Mysore, 345
2. Doneen LD (1964) Water quality for agriculture. Department of Irrigation, University of California, California 48
3. Eaton FM (1950) Significance of carbonate in irrigation waters. Soil Science 69:123–133
4. Hem JD (1985) Study and interpretation of the chemical characteristics of natural water. Department of the Interior, US Geological Survey 2254
5. Raghunath HM (1987) Ground water: hydrogeology, ground water survey and pumping tests, rural water supply and irrigation systems. New Age International
6. Richards LA (1954) Diagnosis and improvement of saline and alkali soils. LWW 78:154
7. Todd DK (1980) Ground water hydrology. Wiley, New York.
8. Wilcox LV (1948) Explanation and interpretation of analyses of irrigation waters. US Department of Agriculture 784
